# Oroxylin A alleviates immunoparalysis of CLP mice by degrading CHOP through interacting with FBXO15

**DOI:** 10.1038/s41598-020-76285-x

**Published:** 2020-11-06

**Authors:** Zhaoxin Zhang, Yun Wang, Yating Shan, Ri Zhou, Wu Yin

**Affiliations:** The State Key Lab of Pharmaceutical Biotechnology, College of Life Sciences, Nanjing University, Nanjing, 210023 China

**Keywords:** Drug discovery, Immunology, Diseases

## Abstract

Clinical reports have found that with the improvement of treatment, most septic patients are able to survive the severe systemic inflammatory response and to enter the immunoparalysis stage. Considering that immunoparalysis leads to numerous deaths of clinical sepsis patients, alleviation of the occurrence and development of immunoparalysis has become a top priority in the treatment of sepsis. In our study, we investigate the effects of oroxylin A on sepsis in cecal ligation and puncture (CLP) mice. We find that the 60 h + 84 h (30 mg/kg) injection scheme of oroxylin A induce the production of pro-inflammatory factors, and further significantly improves the survival of CLP mice during the middle or late stages of sepsis. Mechanistically, C/EBP-homologous protein (CHOP) is upregulated and plays anti-inflammatory roles to facilitate the development of immunoparalysis in CLP mice. Oroxylin A induces the transcription of E3 ligase F-box only protein 15 gene (*fbxo15*), and activated FBXO15 protein binds to CHOP and further mediates the degradation of CHOP through the proteasome pathway, which eventually relieves the immunoparalysis of CLP mice. Taken together, these findings suggest oroxylin A relieves the immunoparalysis of CLP mice by degrading CHOP through interacting with FBXO15.

## Introduction

There are more than 18 million severe sepsis patients worldwide each year, and approximately 14,000 people die every day from sepsis complications, with the incidence of sepsis increasing by 2–7.5% per year^1,2^. According to epidemiological studies, more than half of all deaths in the ICU are caused by sepsis and its complications. Sepsis has exceeded myocardial infarction, becoming the leading cause of death in the ICU. With the development of scientific research, some scholars have found that there are two different immune states during sepsis. The early high inflammatory state can be antagonized by the following compensatory anti-inflammatory response to develop into immunoparalysis^3^. Clinical reports have also found that with the improvement of treatment, most patients were able to survive the severe systemic inflammatory response, and entered into the more complex immunoparalysis stage, and eventually died of secondary nosocomial infections^4^.

Considering that immunoparalysis leads to numerous deaths of clinical patients due to the development of sepsis, many clinical treatments attempt to alleviate immunoparalysis by activating the immunity of the body to some extent during the middle or late stages of clinical sepsis^5–8^. For example, granulocyte–macrophage colony-stimulating factor (GM-CSF), interferon-gamma (IFN-γ), thymosin (Thymosin) and PD-1 antagonists have been proved to enhance immunity and reduce the occurrence and development of sepsis immunoparalysis but still have the defects of large side effects, high dosage, and short half-life^5–8^. Additionally, some clinical and experimental studies have confirmed that many traditional Chinese medicines or their active ingredients usually showed incomparable advantages over western medicine in activating immunity^9–12^.

Recent studies have shown that oroxylin A (5,7-dihydroxy-6-methoxy-2-phenyl-4H-1-benzopyran-4-one, C_16_H_12_O_5_), a natural flavonoid isolated from the traditional Chinese herbs *Scutellariae baicalensis* and *Oroxylum indicum* (Linn.) Kurz, significantly protected mice against LPS-induced acute lung injury or liver injury and improved their survival rate, suggesting that oroxylin A is a potentially useful candidate for the treatment of LPS-induced endotoxaemia and septic shock^13,14^. Rim et al. found that 5,6,7-trimethoxyflavone (TMF), methylations of oroxylin A, increased the survival rate of mice with LPS-induced endotoxaemia^15^. Oroxylin A has been used for centuries as an important herbal medicine in many Asian countries and has been reported to possess a wide spectrum of pharmacologic effects, such as anti-tumour, anti-oxidant, and anti-inflammatory functions^16,17^. However, how oroxylin A inhibits the development of sepsis and whether it is effective for septic patients are still worth exploring.

Interestingly, Hui et al. recently found that oroxylin A negatively regulated the expression of CHOP protein and alleviated retinoic acid syndrome^18^. GADD153/CHOP (growth arrest and DNA damage-inducible gene 153/C/EBP homology protein) is a marker of endoplasmic reticulum stress (ERS) and acts as an intermediate signalling molecule in ERS. Under normal conditions, the expression of *chop* is very low, while in an unfavorable environment such as oxidative stress, starvation or hypoxia, *chop* is activated by ERS and participates in energy metabolism, cell proliferation, differentiation, and apoptosis^19,20^. Additionally, numerous studies have also demonstrated that CHOP usually plays important roles in the regulation of inflammation-related diseases^21–26^. Interestingly, some studies have shown that CHOP usually appeared at higher expression levels in most sepsis models^27–32^. The clinical data also showed that the expression of the *chop* gene in lymphocytes of sepsis patients was significantly upregulated compared with the healthy group; more importantly, the expression of the *chop* gene in the sepsis death group was also significantly higher than that in the survival group^33^. These studies indicate that CHOP may be involved in regulating the development of sepsis. In this study, we found that oroxylin A alleviates the immunoparalysis of CLP mice by degrading CHOP through interacting with FBXO15.

## Results

### The establishment of the CLP mouse model and identification of the immunoparalysis states

To explore the molecular mechanism underlying the development of clinical sepsis, we established the CLP mice model, which has been widely used to study the pathogenesis, pathological processes and treatment of sepsis^34^. In our CLP models, CLP mice began to die at 24 h, and 95% (19 of 20) of the septic mice ultimately died (120 h), mostly from 48–120 h after CLP surgery (12 of 19) (Supplementary Fig. S1a). In contrast with the high mortality, we further found that the bacterial clearance in the blood, peritoneum and spleen of CLP mice at 48–96 h after CLP surgery were much lower than at 0–24 h (Supplementary Fig. S1b).

To explore the reasons underlying the lower bacterial clearance, in consideration of the relevance of cytokines in regulating the development of sepsis^3^, we next evaluated the production of different inflammatory cytokines in serum and peritoneum lavages of CLP mice. The ELISA results showed that the levels of pro-inflammatory cytokines (TNFα, IL-1β and IL-6) were significantly elevated, peaking at 12 h, and then gradually decreasing to the lowest level at 48–96 h after CLP surgery (Supplementary Fig. S1c,d). The production of the anti-inflammatory factor IL-10 gradually increased after CLP surgery and remained at relatively high levels at 48–96 h after CLP surgery (Supplementary Fig. S1c,d). Additionally, the mice blood was collected at 48 h or 72 h after sham or CLP surgery and further stimulated by LPS in vitro, the following ELISA results showed that the production of the TNFα cytokine in the blood of CLP mice presented as an extremely lower level compared with sham mice (Supplementary Fig. S1e). The above results preliminary demonstrated that the CLP mice may have entered a “bacterial or endotoxin tolerance” state at 48–72 h after CLP surgery.

The occurrence and development of immunoparalysis are usually accompanied by shifts in the different types of leukocyte populations in different tissues and organs of sepsis patients. Typically, reductions in CD4^+^ and CD8^+^ T cells, especially the ratio of CD4^+^/CD8^+^ cells have been identified as hallmarks of immunoparalysis^35^. With flow cytometric analysis, we found that the percentages of CD4^+^ and CD8^+^ T cells in total spleen cells and the ratio of CD4^+^/CD8^+^ cells at 48 and 72 h after CLP surgery were all significantly lower than at 0 or 12 h after CLP surgery (Supplementary Fig. S1f,g). Among them, the ratios of spleen CD4^+^/CD8^+^ cells at 48 or 72 h after CLP surgery were 0.97 and 1.03, respectively, while the ratio at 0 h was 1.40 (Supplementary Fig. S1g). In summary, we speculated that CLP mice might switch into an immunoparalysis state at 48–72 h after surgery, and most CLP mice eventually died of aggravated bacterial leakage or propagation during immunoparalysis.

### Oroxylin A alleviates immunoparalysis and improves the survival rate of CLP mice

**Figure 1:**
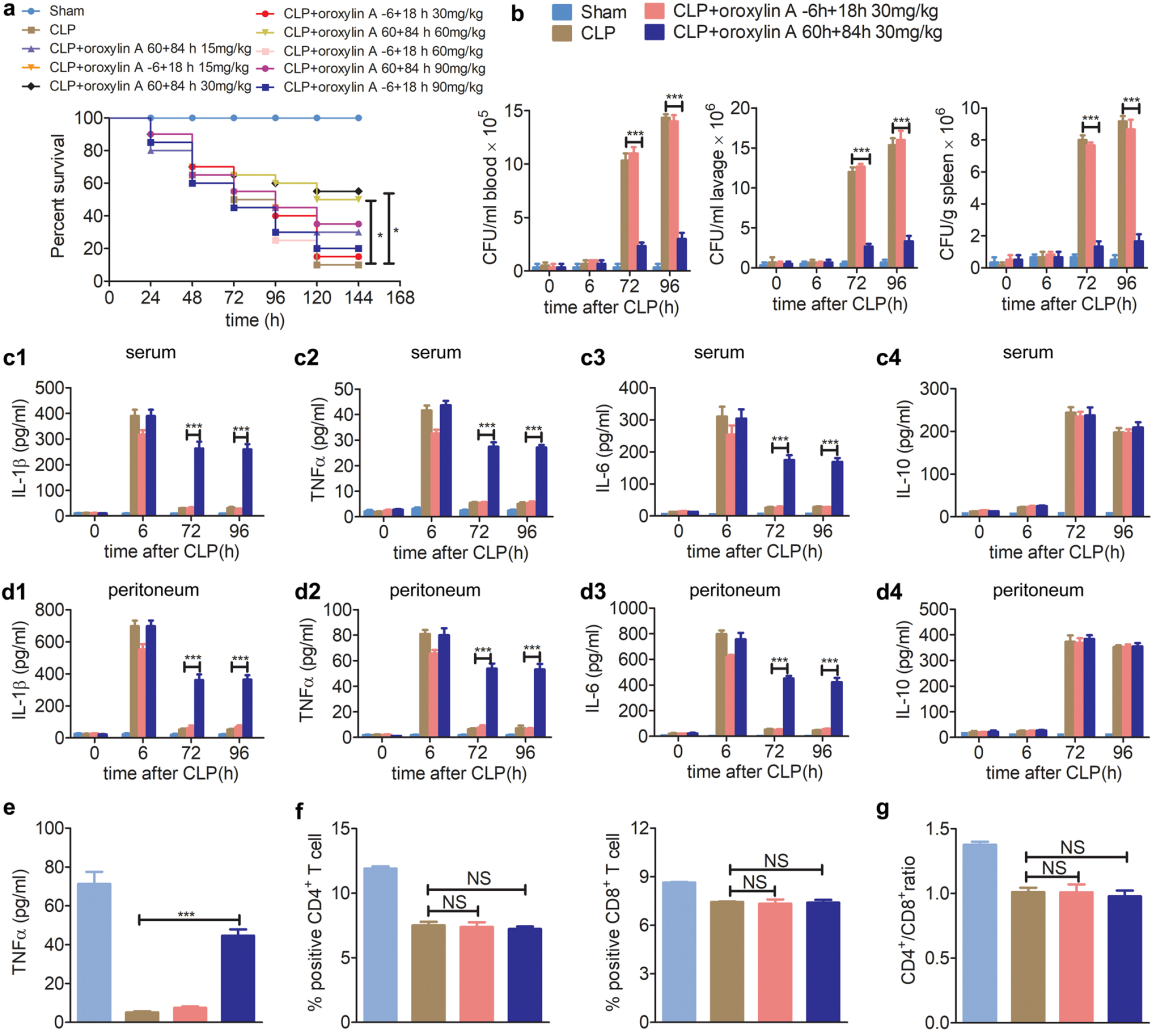
Oroxylin A alleviates immunoparalysis in CLP mice. (**a**) Mice were injected with oroxylin A (15, 30, 60, 90 mg/kg, i.p.) at the indicated times, and the effect of oroxylin A on the mortality of CLP mice was detected (n = 20). Kaplan–Meier curves and the log-rank test were used to compare mortality rates, *p < 0.05. (**b**) The effects of oroxylin A (30 mg/kg) on the bacterial clearance in blood, peritoneum lavage and spleen of CLP mice (n = 6). (**c**) The effects of oroxylin A (30 mg/kg) on the production of IL-1β (**c1**), TNFα (**c2**), IL-6 (**c3**) and IL-10 (**c4**) in serum were examined at the indicated times after CLP by ELISA (n = 6). (**d**) The effects of oroxylin A (30 mg/kg) on the production of IL-1β (**d1**), TNFα (**d2**), IL-6 (**d3**) and IL-10 (**d4**) in peritoneum lavage were examined at the indicated times after CLP by ELISA (n = 6). (**e**) Blood was collected at 96 h after CLP and stimulated with LPS (100 ng/ml) for 1 h. The level of TNFα was examined by ELISA (n = 3). (**f**) The percentages of CD4^+^ and CD8^+^ T cells in the spleen were examined at 96 h after CLP by flow cytometry (n = 3). The flow histograms are presented in Supplementary Figs. S17 and S18. (**g**) The ratio of CD4^+^/CD8^+^ T cells in the spleen was calculated according to the corresponding percentages of CD4^+^ and CD8^+^ T cells, respectively (n = 3). The data are represented as the mean ± SD (**b**–**g**), NS > 0.05 and ***p < 0.001, Student’s t test.

To explore whether oroxylin A plays a role in the treatment of CLP mice, male C57BL6 mice were divided into 10 groups as follows: sham group, CLP group, mice were injected twice with oroxylin A (15, 30, 60, 90 mg/kg, i.p.) at 6 h before CLP and at 18 h after CLP surgery, and mice were injected twice with oroxylin A (15, 30, 60, 90 mg/kg, i.p.) at 60 h and 84 h after CLP surgery. We found that the − 6 h + 18 h injection scheme did not effectively improve the survival of CLP mice. However, the 60 h + 84 h injection scheme, especially 30 mg/kg, significantly improved CLP mice survival rate at 120 h (55% of CLP mice survived) (Fig. 1a). These results suggest that injection with oroxylin A during immunoparalysis is much more effective than injection at the early hyper-inflammatory state.

Next, we explored the molecular mechanism by which oroxylin A improved the survival rates of CLP mice during the middle or late stages of sepsis. We found that the 60 h + 84 h (30 mg/kg) injection scheme significantly improved bacterial clearance in the blood, peritoneum and spleen at 72 h and 96 h after CLP surgery (Fig. 1b). We further evaluated the effect of oroxylin A on the levels of the inflammatory cytokines IL-1β, TNFα, IL-6 and IL-10 in the serum and peritoneum of septic mice by ELISA. Although these pro-inflammatory cytokines (TNFα, IL-1β and IL-6) had decreased to the lowest level at 48–96 h after CLP surgery, the 60 h + 84 h (30 mg/kg) injection scheme of oroxylin A significantly reversed the decreasing trends and maintained them at higher levels at 72 h and 96 h after CLP (Fig. 1c,d). Conversely, the − 6 h + 18 h (30 mg/kg) injection scheme could decrease the levels of pro-inflammatory factors to a lesser extent at 6 h after CLP surgery (corresponding to the hyper-inflammatory state) but had no effect on the levels of pro-inflammatory factors at 72 h and 96 h after CLP surgery. Additionally, two different oroxylin A injection schemes both had no effect on the level of the anti-inflammatory factor IL-10 after CLP surgery (Fig. 1c,d). Additionally, the mice blood was collected at 96 h after sham or CLP surgery and further stimulated by LPS in vitro. The following ELISA results showed that the 60 h + 84 h (30 mg/kg) injection scheme of oroxylin A could remarkably elevate the production of the TNFα cytokine in the blood of CLP mice (Fig. 1e).

We further evaluated the effect of oroxylin A on the percentages of CD4^+^ and CD8^+^ T cells in total spleen cells by flow cytometry. Compared to the sham mice, the percentages of CD4^+^ and CD8^+^ T cells in total spleen cells of CLP mice, the ratio of CD4^+^/CD8^+^ cells were all much lower at 96 h after CLP surgery. But interestingly, the 60 h + 84 h and − 6 h + 18 h (30 mg/kg) injection schemes both nearly had no effect on the percentages of CD4^+^, CD8^+^ T cells and the ratio of CD4^+^/CD8^+^ cells in spleen of CLP mice (Fig. 1f,g). These results suggest that oroxylin A could markedly alleviate the immunoparalysis of CLP mice and further improves the survival of CLP mice by increasing the production of pro-inflammatory factors, but not through regulating the percentages of T cells.

### Knockdown of *chop* improves the survival rate of CLP mice

Numerous studies have shown that the expression of *chop* is significantly upregulated in a variety of sepsis models^27–32^ or clinical research studies^33^. Monocytes/macrophages are sentinel cells of the innate immune system, while lymphocytes are the main adaptive immune cells. Additionally, they have been identified to play very important roles throughout all phases of sepsis and to affect both immune homeostasis and inflammatory processes. In this research, we also found that the expression levels of CHOP in peritoneal macrophages, peripheral blood mononuclear cells (PBMCs) and splenic lymphocytes of CLP mice were all elevated and remained at high levels during immunoparalysis (48–96 h after CLP surgery) (Supplementary Fig. S2). In summary, we preliminarily speculated that CHOP might be involved in regulating the development of sepsis.

**Figure 2:**
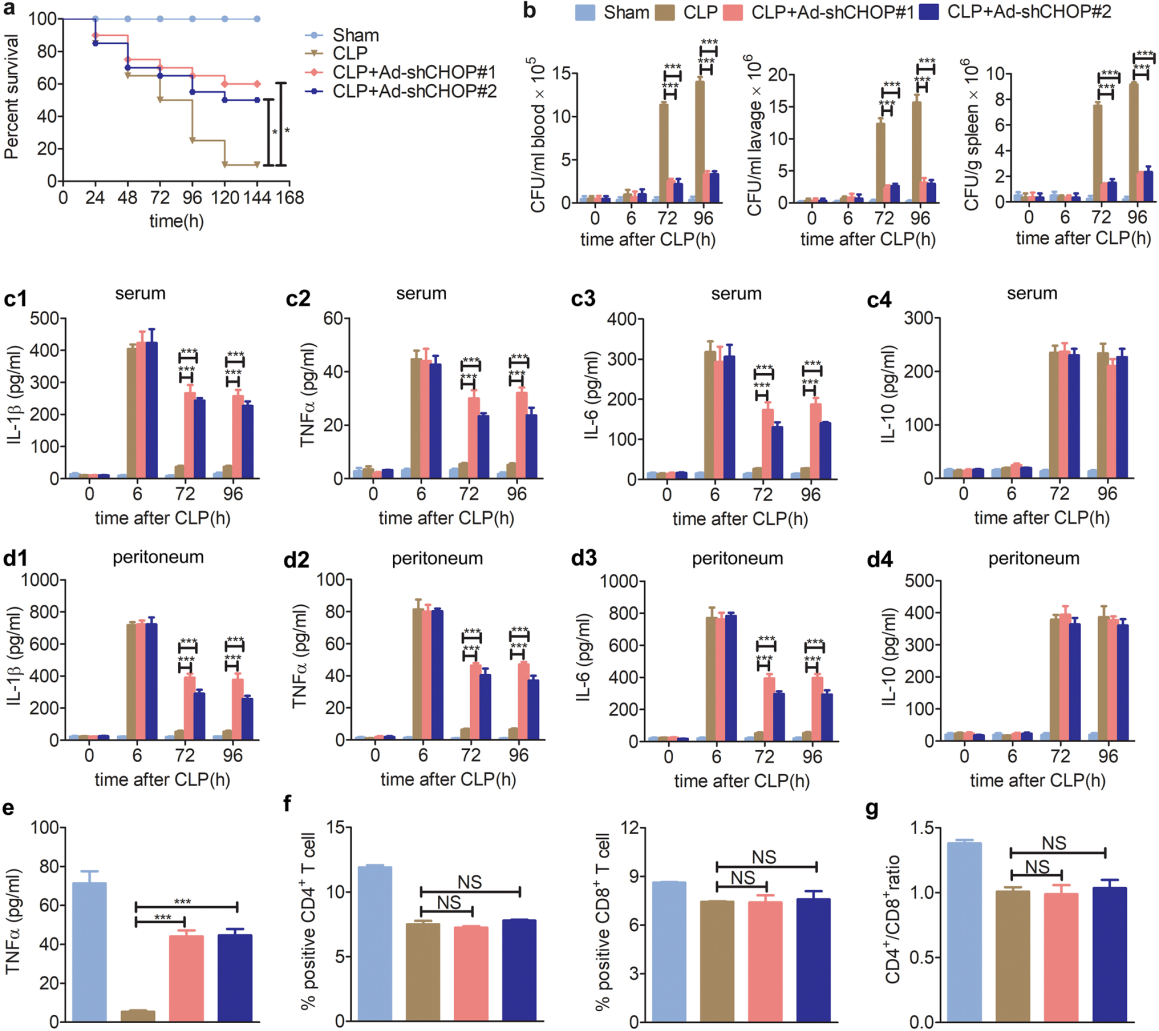
Knockdown of *chop* alleviates immunoparalysis in CLP mice. (**a**) Adenoviruses expressing shRNAs specifically targeting *chop* were intravenously injected into C57BL6 mice through the tail vein. After 7 days, CLP was performed. Effects of *chop*-knockdown on the mortality of CLP mice was detected (n = 20), Kaplan–Meier curves and the log-rank test were used to compare mortality rates, *p < 0.05. (**b**) The effects of *chop*-knockdown on the bacterial clearance in blood, peritoneum lavage and spleen of CLP mice were examined at the indicated times after CLP (n = 6). (**c**) The effects of *chop*-knockdown on the production of IL-1β (c1), TNFα (c2), IL-6 (c3) and IL-10 (c4) in serum were examined at the indicated times after CLP by ELISA (n = 6). (**d**) The effects of *chop*-knockdown on the production of IL-1β (d1), TNFα (d2), IL-6 (d3) and IL-10 (d4) in peritoneum lavage were examined at the indicated times after CLP by ELISA (n = 6). (**e**) Blood was collected at 96 h after CLP and stimulated with LPS (100 ng/ml) for 1 h. The level of TNFα was examined by ELISA (n = 3). (**f**) The percentages of CD4^+^ and CD8^+^ T cells in the spleen were examined at 96 h after CLP by flow cytometry (n = 3). The flow histograms are presented in Supplementary Figs. S19 and S20. (**g**) The ratio of CD4^+^/CD8^+^ T cells in the spleen was calculated according to the corresponding percentages of CD4^+^ and CD8^+^ T cells, respectively (n = 3). The data are represented as the mean ± SD (**b**–**g**), NS > 0.05 and ***p < 0.001, Student’s t test.

To investigate the role of highly expressed CHOP in sepsis, especially during immunoparalysis, C57BL6 mice were injected with adenoviruses expressing shRNAs specifically targeting *chop* through the tail vein. One week later, sham and CLP surgeries were carried out. The two shRNAs could effectively knockdown the high expression levels of *chop* mRNA in peritoneal macrophages, PBMCs and splenic lymphocytes of CLP mice at different time points (Supplementary Fig. S3). In further research, we found that 60% (shCHOP#1) or 50% (shCHOP#2) of the septic mice with knockdown of the *chop* gene survived compared with 10% of control CLP septic mice (Fig. 2a).

Mechanistically, we further found that knockdown of *chop* improved bacterial clearance in blood, peritoneum and spleen during immunoparalysis (72 h and 96 h after CLP) (Fig. 2b), and consistently, knockdown of *chop* significantly reversed the decreases in IL-1β, TNFα and IL-6 expression levels during immunoparalysis (72 h and 96 h after CLP) but had no effect on the level of the anti-inflammatory factor IL-10 (Fig. 2c,d). Additionally, the blood was collected at 96 h after sham or CLP surgery and further stimulated by LPS in vitro. The following ELISA results showed that knockdown of *chop* could remarkably elevate the production of the TNFα cytokine in the blood of CLP mice (Fig. 2e). In addition, further flow cytometric analysis showed that the percentages of spleen CD4^+^ and CD8^+^ T cells and the ratio of CD4^+^/CD8^+^ nearly had no difference in *chop-*knockdown and WT mice at 96 h after CLP surgery (Fig. 2f,g). Therefore, we speculated that CHOP might not involved in regulating the population of CD4^+^ and CD8^+^ T cells in the development of CLP sepsis. In summary, CHOP may negatively regulate the production of pro-inflammatory factors and further facilitates the development of immunoparalysis. In contrast, knockdown of *chop* alleviates immunoparalysis and further improves the survival rate of CLP mice.

### Oroxylin A alleviates immunoparalysis through facilitating the proteasomal degradation of CHOP

**Figure 3:**
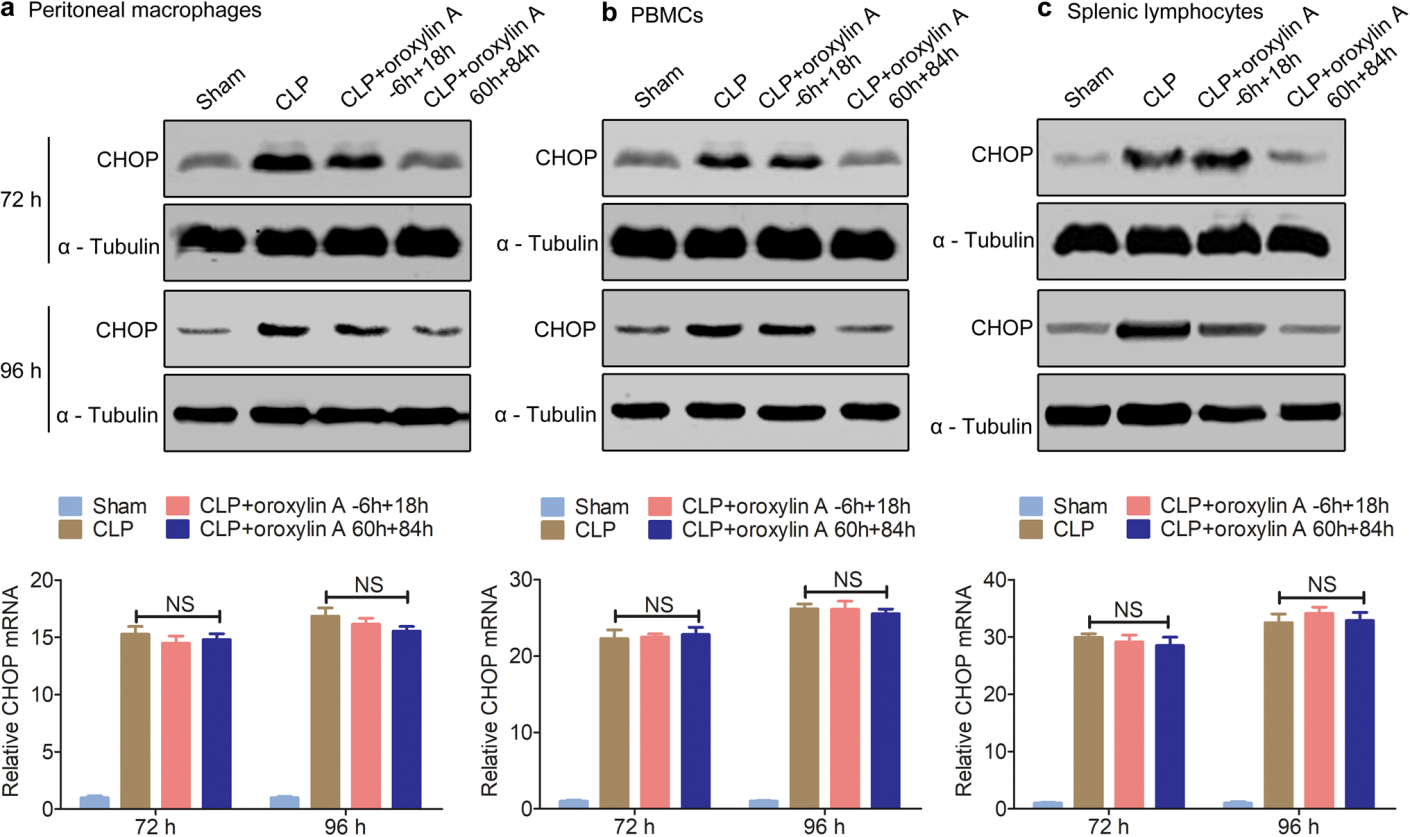
Oroxylin A downregulates the protein level of CHOP during immunoparalysis. CLP mice were injected with oroxylin A (30 mg/kg) at the indicated times, and the protein and mRNA levels of CHOP in peritoneal macrophages (**a**), PBMCs (**b**) and splenic lymphocytes (**c**) were examined at 72 h and 96 h by qPCR and western blotting (n = 6). The data are represented as the mean ± SD, NS > 0.05, Student’s t test. The full-length blots are presented in Supplementary Fig. S11.

Hui et al.^18^ found that in retinoic acid syndrome, oroxylin A can negatively regulate the expression of CHOP protein, thereby alleviating the disease. Interestingly, we also found that the survival of *chop*-knockdown CLP mice was not further improved by oroxylin A injection (Supplementary Fig. S4). Thus, we speculated that the *chop* gene may be the target of oroxylin A in CLP septic mice. Without oroxylin A, the mRNA and protein levels of CHOP were upregulated and remained at high levels during immunoparalysis (48–96 h after CLP surgery) in peritoneal macrophages, PBMCs and splenic lymphocytes (Supplementary Fig. S2). However, the 60 h + 84 h oroxylin A injection scheme significantly reduced the protein levels of CHOP at 72 h and 96 h after CLP surgery (corresponding to the immunoparalysis state) in peritoneal macrophages, PBMCs and splenic lymphocytes (Fig. 3), but the mRNA levels of *chop* remained nearly unchanged (Fig. 3). In addition, we found that the − 6 h + 18 h injection scheme had almost no effect on the level of CHOP protein at 72 h and 96 h after CLP surgery (Fig. 3). We speculated that oroxylin A may negatively regulate the expression of the *chop* gene at a post-transcriptional level during immunoparalysis.

**Figure 4:**
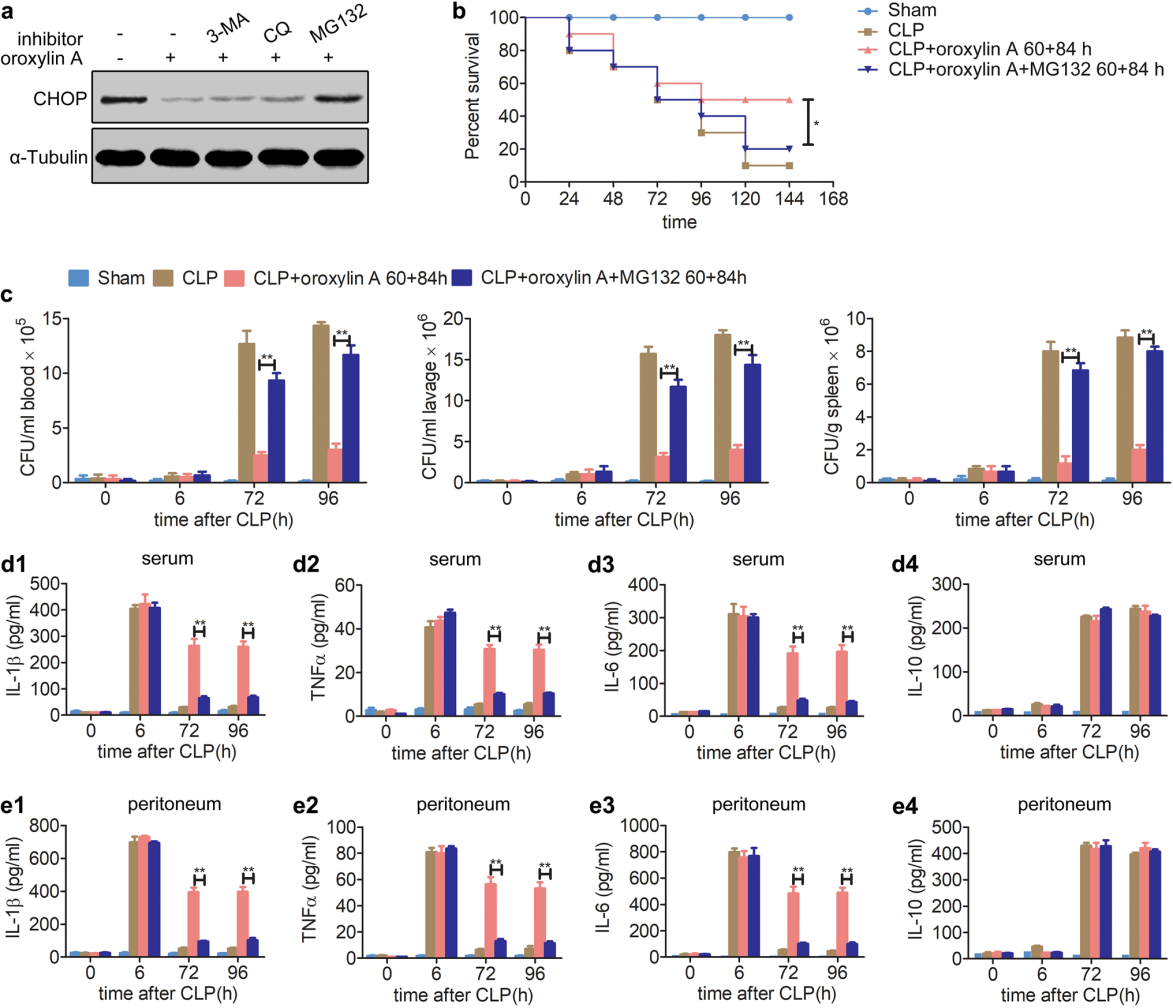
Oroxylin A mediates the proteasomal degradation of CHOP protein. (**a**) CLP mice were intraperitoneally injected with 3-MA (30 mg/kg), CQ (60 mg/kg) or MG132 (3 mg/kg) combined with oroxylin A (30 mg/kg) at 60 h and 84 h after CLP. The protein level of CHOP in peritoneal macrophages was examined at 96 h after CLP by western blotting. The full-length blots are presented in Supplementary Fig. S12. (**b**) MG132 inhibited oroxylin A's improvement in survival rate of CLP mice (n = 10). Kaplan–Meier curves and the log-rank test were used to compare mortality rates, *p < 0.05. (**c**) MG132 inhibited oroxylin A's improvements in the bacterial clearance in blood, peritoneum lavage and spleen of CLP mice (n = 6). (**d**) MG132 inhibited oroxylin A's improvements in serum IL-1β (**d1**), TNFα (**d2**) and IL-6 (**d3**) levels, and had no effect on the level of IL-10 (**d4**) in CLP mice during immunoparalysis (n = 6). (**e**) MG132 inhibited oroxylin A's improvements in peritoneum lavage IL-1β (**e1**), TNFα (**e2**) and IL-6 (**e3**) levels, and had no effect on the level of IL-10 (**e4**) in CLP mice during immunoparalysis (n = 6). The data are represented as the mean ± SD (**c**–**e**), **p < 0.01 Student’s t test.

We next explored whether oroxylin A negatively regulates the expression of CHOP protein through a protein degradation pathway. Since protein degradation mainly depends on the autophagy-lysosomal and ubiquitin–proteasome pathways^36,37^, we further studied the mechanisms underlying how oroxylin A regulates the degradation of CHOP protein. CLP mice were intraperitoneally injected with the autophagy inhibitor 3-MA, CQ or the proteasome inhibitor MG132 combined with oroxylin A (30 mg/kg) twice at 60 h and 84 h after CLP. Western blot analysis showed that oroxylin A reduced the protein level of CHOP at 96 h after CLP surgery (Fig. 4a), but MG132 treatment could significantly resume CHOP protein levels, while the autophagy inhibitor 3-MA or CQ had no similar effect (Fig. 4a). The above results suggested that the proteasome inhibitor MG132 inhibited the degradation of CHOP protein induced by oroxylin A. Therefore, we speculated that oroxylin A may facilitate the proteasomal degradation of CHOP during immunoparalysis with an as-yet unknown mechanism.

Previous experiments have confirmed that oroxylin A may facilitate the degradation of CHOP by the proteasome pathway, and CHOP has been demonstrated to play an important role in the development of CLP sepsis. We then further explored whether oroxylin A improves the survival of CLP mice by degrading CHOP during immunoparalysis. Mice were injected with MG132 (3 mg/kg, i.p.) and oroxylin A twice at 60 h and 84 h after CLP. Remarkably, we found that MG132 inhibited the improved effect of oroxylin A on the survival of CLP mic (Fig. 4b). We further found that MG132 inhibited the improved effect of oroxylin A on the bacterial clearance in blood, peritoneum and spleen at 72 h and 96 h after CLP surgery (Fig. 4c). Consistently, the improvement effects mediated by oroxylin A on cytokine production in the serum and peritoneum were also inhibited by MG132 at 72 h and 96 h after CLP surgery (Fig. 4d,e). Additionally, the mice blood was collected at 96 h after sham or CLP surgery and further stimulated by LPS in vitro. The following ELISA results showed that the improvement effect of oroxylin A (the 60 h + 84 h injection scheme) on TNFα production in the blood of CLP mice was also inhibited by MG132 (Supplementary Fig. S5). These results indicate that oroxylin A alleviates the immunoparalysis of CLP mice by mediating CHOP degradation in a proteasome-dependent manner.

### CHOP interacts with the E3 ubiquitin ligase FBXO15

**Figure 5:**
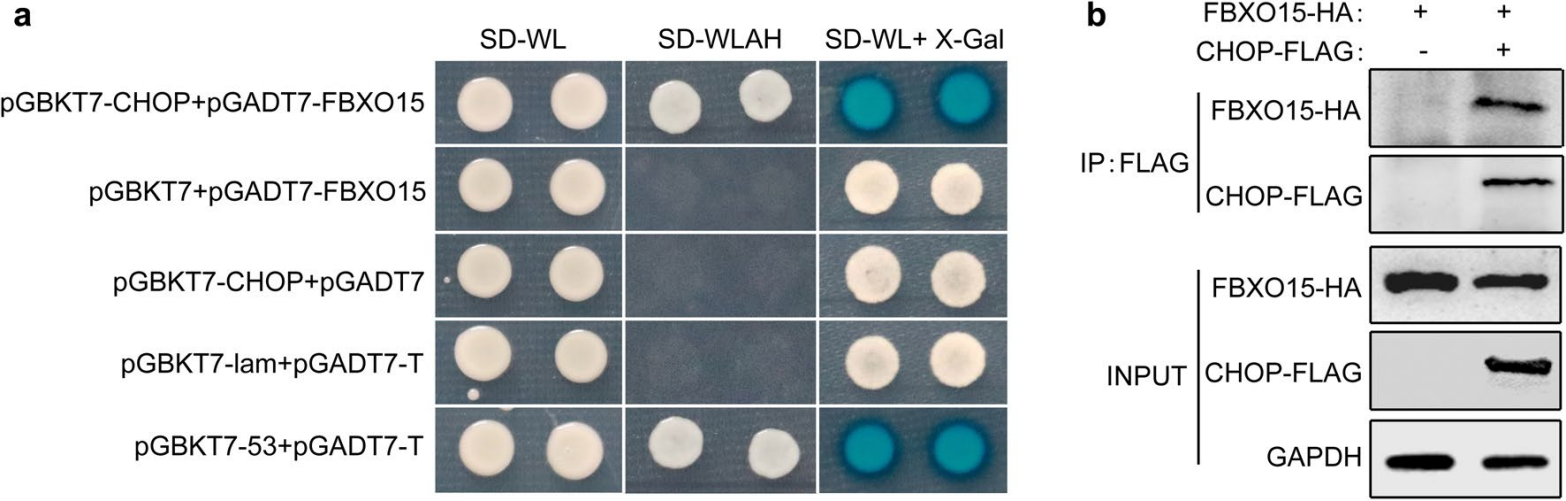
CHOP interacts with E3 ubiquitin ligase FBXO15. (**a**) Yeast two-hybrid assay for the interaction between CHOP and FBXO15. Protein–protein interactions were examined by cell growth on SD medium lacking Trp and Leu (SD-WL) and SD medium lacking Trp, Leu, Ade and His (SD-WLAH) plates. The colonies from the SD-WL plates were further assayed for β-galactosidase activity with an overlay assay. The transformants expressing pGBKT7-53 and pGADT7-T were used as positive controls, and the transformants expressing pGBKT7-lam and pGADT7-T were used as negative controls. (**b**) Raw264.7 cells were transfected with a CHOP-FLAG expression plasmid together with a FBXO15-HA expression plasmid. Twenty-four hours later, the cells were treated with MG132 (2 μM) for 24 h and subjected to immunoprecipitation with an anti-FLAG antibody followed by immunoblotting with anti-HA and anti-FLAG antibodies. The full-length blots are presented in Supplementary Fig. S12.

Although oroxylin A plays a protective role in sepsis by degrading CHOP as described above, the mechanism underlying this effect has not been well characterized. We further screened for proteins that interact with CHOP using a yeast two-hybrid (Y2H) cDNA library from mice. First, the *chop* gene was cloned into the pGBKT7 vector as a bait. Interestingly, after yeast growth screening and further sequencing of different clones, FBXO15 (GenBank accession number NM_001367965.1) was identified as a prominent candidate. FBXO15 is a substrate recognition component of a SCF (SKP1-CUL1-F-box protein) E3 ubiquitin-protein ligase complex that mediates the ubiquitination and subsequent proteasomal degradation of target proteins. We further confirmed the interaction between CHOP and FBXO15 full-length protein by paired Y2H tests. Using multiple controls, reporter gene activation confirmed the interaction between these two proteins in yeast (Fig. 5a). Next, a co-immunoprecipitation assay in which FBXO15-HA was transiently expressed together with CHOP-FLAG revealed that FBXO15 bound to CHOP in the presence of MG132 (Fig. 5b). These results indicate that CHOP specifically interacts with E3 ubiquitin ligase FBXO15.

### Oroxylin A upregulates the expression of FBXO15 during immunoparalysis

**Figure 6:**
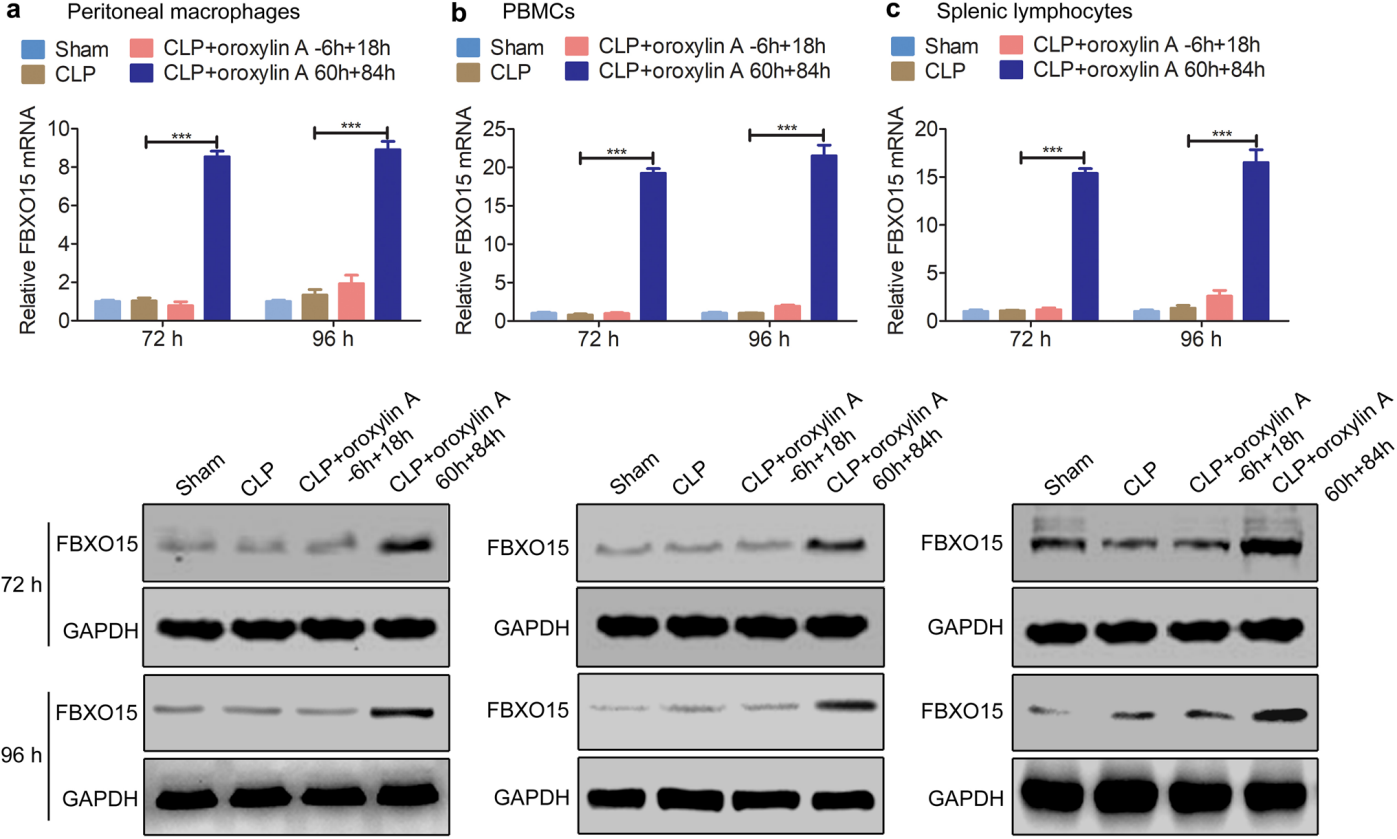
Oroxylin A upregulates FBXO15 during immunoparalysis. CLP mice were injected with oroxylin A (30 mg/kg) at the indicated times and the mRNA and protein levels of FBXO15 in peritoneal macrophages (**a**), PBMCs (**b**) and splenic lymphocytes (**c**) were examined at 72 h and 96 h by qPCR and western blotting. The data are represented as the mean ± SD, ***p < 0.001, Student’s t test. The full-length blots are presented in Supplementary Figs. S13.

We next explored whether there is a definite association between oroxylin A and FBXO15. First, we found that the mRNA and protein expression levels of FBXO15 were extremely low in peritoneal macrophages, PBMCs and splenic lymphocytes of CLP mice, similar to sham mice at 72 h and 96 h after surgery (Fig. 6). However, 60 h + 84 h (30 mg/kg) injection of oroxylin A significantly increased the mRNA and protein levels of FBXO15 in these cells at 72 h and 96 h after surgery (Fig. 6). Moreover, oroxylin A treatment also induced the expression of *fbxo15* gene in these cells in vitro (Supplementary Fig. S6). These results indicate that oroxylin A could significantly induce the expression of *fbxo15* gene at a transcriptional level.

### Knockdown of *fbxo15* inhibits the efficacy of oroxylin A on sepsis

**Figure 7:**
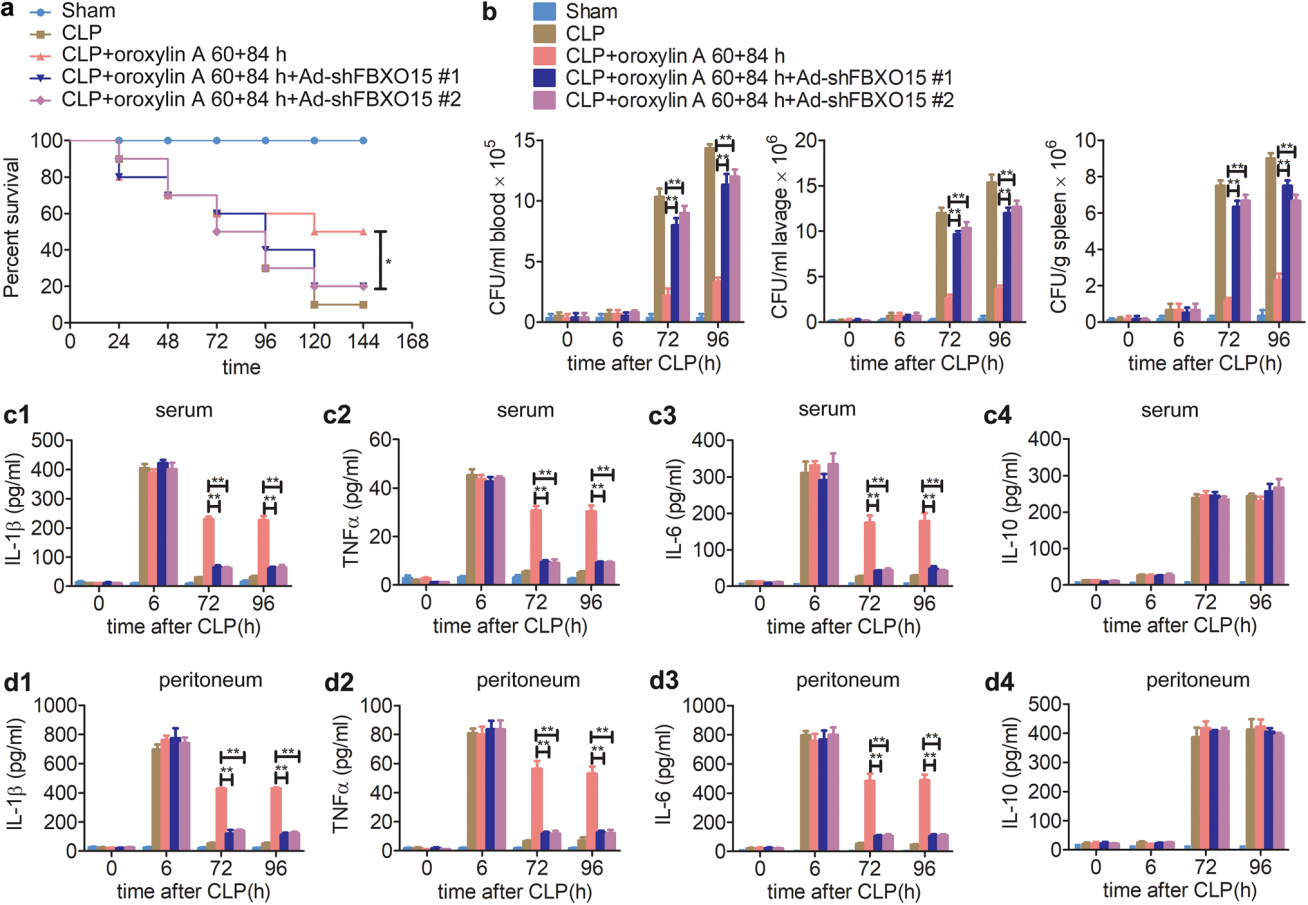
Knockdown of *fbxo15* inhibits the efficacy of oroxylin A on sepsis. Adenoviruses expressing shRNAs specifically targeting *fbxo15* were intravenously injected into C57BL6 mice through the tail vein. After 7 days, CLP was performed and oroxylin A (30 mg/kg) was injected at 60 h and 84 h after CLP. (**a**) Knockdown of *fbxo15* inhibited oroxylin A's improvement in survival rate of CLP mice (n = 10). Kaplan–Meier curves and the log-rank test were used to compare mortality rates, *p < 0.05. (**b**) Knockdown of *fbxo15* inhibited oroxylin A's improvements in the bacterial clearance in blood, peritoneum lavage and spleen of CLP mice (n = 6). (**c**) Knockdown of *fbxo15* inhibited oroxylin A's improvements in serum IL-1β (**c1**), TNFα (**c2**) and IL-6 (**c3**) levels, and had no effect on the level of IL-10 (**c4**) in CLP mice during immunoparalysis (n = 6). (**d**) Knockdown of *fbxo15* inhibited oroxylin A's improvements in peritoneum lavage IL-1β (**d1**), TNFα (**d2**) and IL-6 (**d3**) levels, and had no effect on the level of IL-10 (**d4**) in CLP mice during immunoparalysis (n = 6). The data are represented as the mean ± SD (**b**–**d**), **p < 0.01, Student’s t test.

Since FBXO15 binds to CHOP, we further explored whether FBXO15 plays a role in regulating the degradation of CHOP mediated by oroxylin A. C57BL6 mice were injected with adenoviruses expressing shRNAs specifically targeting *fbxo15* through the tail vein. One week later, sham and CLP surgeries were carried out. The two shRNAs could effectively knockdown the high expression levels of *fbxo15* mRNA in peritoneal macrophages, PBMCs and splenic lymphocytes of CLP mice at 96 h after surgery (Supplementary Fig. S7). Western blotting revealed that 60 h + 84 h (30 mg/kg) injection of oroxylin A facilitated the degradation of CHOP at 96 h after surgery, but the degradation was blocked by *fbxo15* knockdown (Supplementary Fig. S8). More importantly, we further found that knockdown of *fbxo15* inhibited oroxylin A's improvement on the survival of CLP mice (Fig. 7a).

Mechanistically, we further found that knockdown of *fbxo15* inhibited the improved effect of oroxylin A on the bacterial clearance in blood, peritoneum and spleen at 72 h and 96 h after CLP surgery (Fig. 7b). Consistently, the improvements of oroxylin A on cytokine production in the serum and peritoneum were also inhibited by knockdown of *fbxo15* at 72 h and 96 h after CLP surgery (Fig. 7c,d). Additionally, the blood was collected at 96 h after sham or CLP surgery and further stimulated by LPS in vitro. The following ELISA results showed that the improvement effect of oroxylin A (the 60 h + 84 h injection scheme) on TNFα production in the blood of CLP mice was also inhibited by knockdown of *fbxo15* (Supplementary Fig. S9). These results indicate that knockdown of *fbxo15* blocks the degradation of CHOP and further inhibits the efficacy of oroxylin A on sepsis.

## Discussion

Oroxylin A, a natural flavonoid isolated from the traditional Chinese herbs *Scutellariae baicalensis* and *Oroxylum indicum* (Linn.) Kurz, has been reported to possess a wide spectrum of pharmacologic activities, such as antitumor, antioxidant, and anti-inflammatory functions^16,17^. Recent studies have found that oroxylin A exerts a broad anti-inflammatory effect in different inflammatory diseases. Jung et al*.* found that oroxylin A significantly inhibited the production of NO, thereby inhibiting the activation of NF-κB and the expression of inflammatory factors in LPS-stimulated peritoneal macrophages^38^. Yao et al. found that oroxylin A inhibited NF-κB p65 nuclear translocation and phosphorylation of IκBα and IKKα/β and suppressed LPS-induced pro-inflammatory cytokine secretion in human monocyte THP-1 cells^39^. Consistently, in our study, ELISA results also showed that the − 6 h + 18 h (30 mg/kg) injection scheme of oroxylin A could decrease the levels of pro-inflammatory factors to a lesser extent at the early stage of CLP sepsis (6 h after CLP surgery, corresponding to the hyper-inflammatory state), but its effect on improving the survival of CLP mice was extremely limited (i.e., there was almost no effect) (Fig. 1). In summary, suppression of inflammatory storms in the early stage of sepsis and reduction of excessive inflammatory damage have extremely limited effects on improving the survival of CLP mice.

In contrast, the 60 h + 84 h (30 mg/kg) injection scheme of oroxylin A (corresponding to the immunoparalysis state) could effectively restore the immunity and further greatly enhance the survival rate of CLP mice by improving the production of pro-inflammatory cytokines (Fig. 1). In summary, we speculated that the improvement effect of oroxylin A on the survival of CLP mice mainly depends on the activation of immunity and further alleviating the immunoparalysis in the middle and late stages of sepsis. Based on above studies, we further speculated that oroxylin A could play a dual role in regulating the development of inflammation (pro-inflammatory or anti-inflammatory), and relying on different regulatory mechanisms. oroxylin A may usually play anti-inflammatory effects in hyper-inflammatory state, while acts as a pro-inflammatory role in hypo-inflammatory state. And the molecular mechanism underlying how oroxylin A play anti-inflammatory or pro-inflammatory role in different inflammation conditions is still unclear, but we demonstrated the pro-inflammatory role of oroxylin A may be closely related to ERS marker CHOP in sepsis of CLP mice.

In a recent study, Rim et al. found that TMF, methylations of oroxylin A, increased the survival rate of mice with LPS-induced endotoxemia^15^. In addition, we also found that TMF could improve the survival of CLP mice to a certain extent when TMF was injected twice at − 6 h and 18 h after CLP surgery. However, the improvement range of the survival of CLP mice was significantly lower than that with the 60 h + 84 h injection scheme of TMF (Supplementary Fig. S10). Thus, we speculated that the difference between the effects of TMF and oroxylin A on sepsis might be mainly due to the durations of the two components. TMF is a key intermediate for the synthesis of oroxylin A^40^ and a methylation variant of oroxylin A. Moreover, the methylation of TMF may result in a dramatic increase in metabolic stability through blocking the conjugation pathway of free hydroxyl groups^41^. Thus, we further speculated that TMF could also improve the survival of CLP mice by persistently playing an immune-activating role during the immunoparalysis state due to its longer half-life.

Numerous studies have shown that CHOP is significantly upregulated in a variety of sepsis models^27–32^. CHOP is a marker of ERS and acts as an intermediate signaling molecule in ERS and apoptosis. In endoplasmic reticulum stress, activated CHOP regulates downstream target genes to induce apoptosis, such as downregulating the anti-apoptotic factor Bcl-2 or upregulating the pro-apoptotic factor Bax^42^. CHOP induced apoptosis is involved in the development of various diseases^43,44^, such as neurodegenerative disease, diabetes and acquired immune deficiency syndrome^43,44^. But in this study, we showed that knockdown of *chop* nearly had no effect on the population of CD4^+^ and CD8^+^ T cells in the development of CLP sepsis (Fig. 2). Therefore, we speculated that CHOP might not involved in regulating the apoptosis of immune cells in the development of CLP sepsis.

More importantly, CHOP also plays an important role in the regulation of inflammation-related diseases^21–26^. Some initial studies have found that in a variety of endoplasmic reticulum stress responses, CHOP promotes the development of cellular inflammation^20,45^. However, recent numerous studies have found that CHOP negatively regulates the expression of pro-inflammatory cytokines in the development of sepsis^24–26^. Esposito et al*.* found that mice injected with LPS showed high expression of CHOP in the kidney, and CHOP inhibited the inflammatory response^31^. Consistently, in our study, we found that knockdown of CHOP by shRNAs silencing technology could effectively reduce the expression of *chop*, and further elevate the production of pro-inflammatory cytokines at the middle or late stages of CLP sepsis (Fig. 2). It is true that CHOP knockout could completely delete the expression of *chop* genes in mice (better than CHOP knockdown mice), and have been used in exploring the inflammation-related functions of *chop* gene in some previous studies^24,27,28,31^, but due to some limitations, we did not use CHOP knockout mice in this study. Thus, the functional consistency between CHOP knockout and CHOP knockdown mice in the sepsis-related inflammations requires further exploration.

In addition, we also showed that CHOP had no effect on the level of the anti-inflammatory factor IL-10 in septic mice (Fig. 2). At least, we speculated that CHOP was involved in regulating the production of pro-inflammatory cytokines in an IL-10 independent manner. Thus, the mechanism underlying how CHOP negatively regulates inflammatory factors remains to be further explored. In summary, CHOP may facilitate the occurrence and development of immunoparalysis by negatively regulating the production of pro-inflammatory factors, but not regulating the apoptosis of immune cells in CLP septic mice.

Both knocking down of *chop* and oroxylin A administration improve the survival of CLP mice, is there a regulatory relationship between them? We found that the improved survival of CLP mice that results by knocking down *chop* is not further promoted by treating with oroxylin A (Supplementary Fig. S4). Interestingly, Hui et al*.* found that oroxylin A negatively regulated the expression of CHOP protein and alleviated retinoic acid syndrome^18^. In our study, we found that oroxylin A facilitated the degradation of the CHOP protein during immunoparalysis without affecting the expression of *chop* mRNA (Fig. 3). At present, the most studies about the regulation of CHOP expression mainly focused on transcription levels; while the post-transcriptional regulation s still in infancy^19,20^. Dai et al*.* found that AMPKα1 mediates CHOP ubiquitination and proteasomal degradation in macrophages by promoting the phosphorylation of CHOP at serine 30^46^. In our study, we found that oroxylin A-mediated CHOP degradation in macrophages could be prevented by the proteasome inhibitor MG132 during immunoparalysis (Fig. 4). Further yeast two-hybrid screening indicated that E3 ubiquitin ligase FBXO15 interacted with CHOP (Fig. 5).

The SCF (complex of SKP1, CUL1 and F-box protein) comprises the largest family of E3 ubiquitin ligases in mammals; they mediate substrate ubiquitination and degradation. FBXO15 is a component of F-box proteins, which have been classified into three categories: WD40 domain-containing (FBXWs), LRRs-containing (FBXLs), and other diverse domains-containing (FBXOs). Some studies have shown that F-box protein plays an important role in regulating inflammation^47,48^. In B cell lineages, FBXW7 has a pro-survival role by mediating the degradation of p100, an inhibitor of nuclear factor-κB (NF-κB) signalling^47^. However, no studies showed that FBXO15 have been involved in regulating the inflammation. But some studies have found that FBXO15 is involved in degrading substrates through the ubiquitin–proteasome pathway in other cellular events. Katayama et al*.* found that FBXO15 regulates P-glycoprotein/ABCB1 expression through the ubiquitin–proteasome pathway in cancer cells^49^. Chen et al*.* found that FBXO15 mediated proteasomal degradation of cardiolipin synthase (CLS1) in epithelia, resulting in decreased cardiolipin availability and disrupted mitochondrial function^50^. In our study, we found highly expressed FBXO15 which induced by oroxylin A regulated CHOP degradation and alleviated immunoparalysis. Additionally, knockdown of *fbxo15* blocked the degradation of CHOP (Supplementary Fig. S8) and inhibited the efficacy of oroxylin A on sepsis (Fig. 7), suggesting that the effectiveness of oroxylin A on CLP mice depends on FBXO15. Compared to knockout, knockdown technology cannot completely block the expression of genes, but we found that *fbxo15* shRNAs could effectively reduce the expression of *fbxo15* in CLP mice over a long period of time (Supplementary Fig. S7).

FBXO15 was first proved to be expressed predominantly in mouse undifferentiated embryonic stem (ES) cells and was expressed at extremely lower levels in other cells. A study showed that *fbxo15* is a downstream target of the transcription factor Oct3/4, but homozygous deletion of *fbxo15* had no effect on embryogenesis in mice, nor did it affect the cellular morphology or capacity for proliferation and differentiation of mouse ES cells^51^. Interestingly, we found that *fbxo15* was expressed at extremely low levels in different immune cells of CLP mice, and oroxylin A could significantly upregulate the transcription of *fbxo15* (Fig. 6). However, the molecular mechanism underlying that needs to be further explored.

## Conclusion

**Figure 8:**
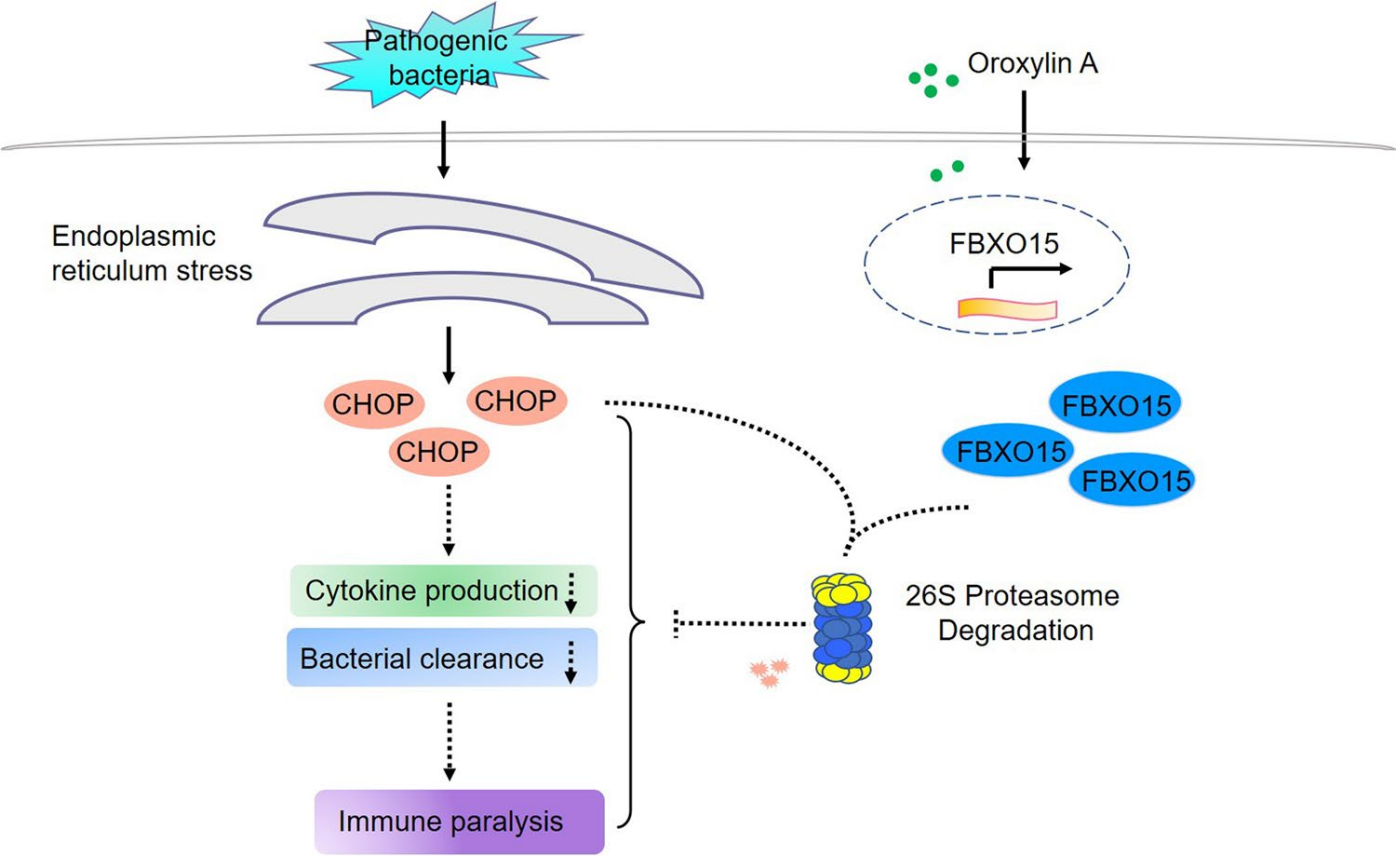
A model illustrating the mechanism by which oroxylin A alleviates immunoparalysis.

The above results are summarized as a model presented in Fig. 8. In CLP mice, in the initial stage of sepsis, macrophages recognize bacterial endotoxin through various PRRs and release large amounts of pro-inflammatory cytokines or activate the adaptive immune response to provide further defense against microbes. With continuous bacterial leakage from the cecal of CLP mice, sustained stimulation of endotoxin could lead to inflammatory responses, ranging from activation to suppression, leading to an immunoparalysis state and resulting in the death of CLP mice from uncontrollable pathogen infection. Mechanistically, continuous pathogen stimulation upregulates CHOP expression through ER stress, and the expression is maintained at high levels during the middle or late stages of sepsis. CHOP negatively regulates the production of pro-inflammatory factors, thereby accelerating and aggravating the occurrence and development of immunoparalysis. Here, we demonstrated that administration of oroxylin A during the middle or late stages of sepsis induces the transcription of the E3 ligase *fbxo15*, which remains at a extremely low level in the absence of oroxylin A. Activated FBXO15 binds to CHOP and further induces CHOP degradation through the proteasome pathway, and further resuming the production of pro-inflammatory factors, eventually alleviating the immunoparalysis of CLP mice. Taken together, these findings suggest that oroxylin A alleviates the immunoparalysis of CLP mice by degrading CHOP through FBXO15.

## Materials and methods

### Chemicals, reagents and antibodies

Dimethyl Sulfoxide (DMSO), LPS (L2630), 3-Methyladenine (3-MA, M9281), Chloroquine (CQ, C6628), and MG132 (M8699) were purchased from Sigma-Aldrich. Oroxylin A and TMF was purchased from Shanghai Yuanye Biotechnology Co., Ltd. IPTG (I3431) was purchased from Technova. ELISA kits for murine TNFα (MTA00B), IL-6 (M6000B), IL-1β (MLB00C) and IL-10 (M1000B) were purchased from R&D Systems. ProteinIso Ni-NTA Resin (DP101) was purchased from TransGene Biotechnology. Protein G Agarose Slurry (20398) was purchased from Pierce Chemical Co. Anti-CHOP antibody (2895) was purchased from Cell Signaling Technology. Anti-FBXO15 (13024-1-AP) antibody, anti-FLAG (66008-3-lg) antibody, anti-HA (51064-2-AP) antibody, anti-Tubulin (11224-1-AP) antibody and anti-GAPDH (60004-1-lg) antibody were purchased from Proteintech Group. FITC anti-mouse CD4 antibody (561828) and PE-Cy5 anti-mouse CD8a antibody (561094) were purchased from BD Pharmingen. TransStart Green qPCR SuperMix (AQ131) was purchased from TransGene Biotechnology (Beijing, China).

### Animals

Male C57BL6 mice (6–8 weeks old) were obtained from the model animal research center of Nanjing University (Nanjing, China) and were housed in standard cages at 25 °C on a 12/12 h light–dark cycle in a clean room and supplied with food and water ad libitum. Cecal ligation and puncture (CLP) were performed as previously described^52^. CLP mice were anesthetized by i.p. injection with 0.1 ml /10 g 4% chloral hydrate. Under sterile conditions, a 1.5-cm incision was created in the lower abdominal region, and the cecum was exposed. The distal portion of the cecum was completely ligated 1 cm from the end with a 3-0 silk suture, punctured once with an 18-gauge needle, and then replaced in the peritoneal cavity. Subsequently, the peritoneal wall and skin were closed with double sutures. Next, s.c. injection of 1 ml of sterile saline (0.9%) was administered to the mice after surgery. Sham mice underwent abdominal incision and cecal exposure without ligation and puncture. After the procedure, mice had access to water and food ad libitum.

Oroxylin A was dissolved in DMSO as a stock solution, stored at − 20 °C, and diluted with saline before experiments. The final DMSO concentration did not exceed 1% throughout the study.

The methods were carried out in accordance with the approved guidelines, and every effort was made to minimize suffering. All experimental protocols were approved by the Animal Experiment Committee of Nanjing University.

### Generation of *chop* knockdown transgenic mice with adenovirus

Adenoviruses expressing shRNAs specifically targeting *chop* were generated as previously described^53^. The sequences are listed in Supplementary Table S1. ShRNAs specific targeting *chop* were cloned into pAd-Track. The resultant plasmid was linearized and subsequently transformed into competent AdEasier cells, which were BJ5183 derivatives containing the adenoviral backbone plasmid pAdEasy-1. Recombinants used for knockdown of *chop* were amplified in HEK293 cells (ATCC) and subsequently purified by CsCl gradient centrifugation. Mice were injected intravenously through the tail vein with the purified adenoviruses at a dose of 1 × 10^9^ PFU per mouse.

### Cell isolation

Peritoneal macrophages were harvested as previously described^54^. Mice were anesthetized and intraperitoneally injected with 5 ml of PBS + 1% BSA, the abdomen was massaged to detach macrophages, and the peritoneal fluid was collected. The cells were allowed to adhere to the substrate by culturing them for 2 h at 37 °C. Then, nonadherent cells were removed by gently washing three times with warm PBS, and the residual adherent cells were macrophages.

Whole blood from mice was collected by removal of the eyeball bleed into EDTA-K2 vacutainer tubes. PBMCs were separated from whole blood using Ficoll/Paque density gradient centrifugation. The PBMC layer was aspirated with pasteur pipettes and washed twice with PBS for subsequent experiments.

Spleens were harvested, cut into pieces and ground. Cell suspension passed through a 70 μm nylon Falcon cell strainer to remove connective tissue and large cell clumps. Residual red blood cells were lysed by hypotonic lysis in ice-cold ammonium chloride and centrifuged at 1500 rpm for 5 min. After washing the pellet with PBS three times, gently added it to the spleen lymphocyte separation solution and centrifuged at 500*g* for 20 min. The lymphocyte layer was aspirated with pasteur pipettes and washed twice with PBS for subsequent experiments.

### Bacterial counts

Blood was collected by direct removal of the eyeball. Peritoneal lavage was performed using 5 ml of sterile PBS. Spleens were harvested using sterile technique, weighed, and homogenized with a sterile tissue homogenizer. Serial dilutions of these samples were plated on tryptic soy agar plates. Bacteria were counted after incubation at 37 °C for 24 h and calculated as CFU per ml of blood or peritoneal fluid.

### Quantitative real-time PCR

This experiment was performed as previously described^55^. Total RNA was extracted from the cells by TRIzol reagent. ReverTra Ace qPCR RT Master Mix was then used to reverse-transcribe the RNA into cDNA, which was then subjected to quantitative PCR with the CFX Connect Real-Time PCR Detection System using TransStart Green qPCR SuperMix under the following conditions: 94 °C for 30 s, then 40 cycles at 94 °C for 5 s, and 60 °C for 30 s. The mRNA levels of specific genes were normalized to β-Actin. The specific primer sequences for *chop* and *fbxo15* are listed in Supplementary Table S1.

### Western blotting

This experiment was performed as previously described^56^. The protein samples were lysed in RIPA buffer for 30 min and then separated by SDS-PAGE. After the electrophoresis, the proteins were transferred from the gel to a PVDF membrane, and then the membrane was placed in 5% milk and blocked at room temperature for 60 min. Next, the blocked membrane was incubated with primary antibody at 4 °C overnight. It was then washed three times with PBST buffer on a shaker for 10 min each. The washed membrane was incubated with a horseradish peroxidase (HRP)-labeled secondary antibody for 1 h. It was then washed three times with PBST buffer on a shaker for 10 min each. Finally, the signal was detected by a Tanon chemiluminescence imaging system.

### Cytokine analysis by ELISA

Mice were injected i.p. with 1 ml of PBS, and peritoneal lavage fluid was collected using a syringe with an attached needle and centrifuged at 400*g* for 10 min at 4 ℃. Supernatant was stored at − 80 ℃ until cytokine measurement. Blood was collected by direct removal of the eyeball, and all samples were kept at room temperature for 1 h and spun at 3000 rpm for 5 min to remove the precipitate. Serum was diluted and assessed for cytokine levels by ELISA according to protocols recommended by the manufacturer.

### Coimmunoprecipitation assay

Cells were lysed in IP buffer and centrifuged at 12,000 rpm for 15 min at 4 °C to remove the insoluble fraction. For immunoprecipitation, 0.5 ml of the supernatant was incubated with 0.5 μg of antibody plus 30 μl of a 50% slurry of protein G agarose at 4 °C overnight. The immunoprecipitates were washed three times, resuspended in 20 μl of SDS loading buffer and subjected to immunoblotting.

### Yeast two-hybrid (Y2H) screening systems

Y2H techniques were performed according to the Matchmaker GAL4 Two-hybrid System 3 manual as described earlier^57,58^. The entire *chop* coding region was amplified by PCR using the primers listed in Supplementary Table S1 and inserted in the pGBKT7 vector generating a fusion with the GAL4 DNA binding domain (BD). The yeast AH109 strain carrying the pGBKT7-CHOP plasmids was mated with Y187 cells pretransformed with a mouse thymus cDNA library (GeneCreate Biotech). Cells were selected on SD medium lacking Leu and Trp (SD-WL) and SD medium lacking Leu, Trp, Ade, and His (SD-WLAH). Library plasmids from positive clones of SD-WLAH were isolated from yeast cells and transformed into *E. coli* DH5a and the cDNA inserts were sequenced. Direct interaction of two proteins was investigated by co-transformation of the pGBKT7-CHOP and pGADT7-FBXO15 plasmids in the yeast strain AH109, followed by selection of transformants on SD-WL plates at 28 ℃ for 3 days and subsequent transfer to SD-WLAH plates for growth selection. Yeasts growing on SD-WL medium were further overlaid with X-Gal solution (0.5 M phosphate buffer [pH 7.0], 7% *N*,*N*-dimethylformamide, 0.5% agarose, 0.1% SDS, 0.4 mg/ml 5-bromo-4-chloro-3-indolyl-β-d-galactopyranoside [X-Gal]) to test the lacZ activity as described earlier.

### Flow cytometric analysis

This experiment was performed as previously described^59^. Spleens were harvested, and the cells were suspended by gentle grinding and passed through a 70 μm nylon Falcon cell strainer to remove connective tissue and large cell clumps. Residual red blood cells were lysed by hypotonic lysis in ice-cold ammonium chloride. Cells were then washed twice in PBS. Total cell counts were obtained using the Invitrogen Countess Automated Cell Counter. The cells were resuspended in PBS at a concentration of 5 × 10^6^ cells/ml. The percentages of CD4^+^ or CD8^+^ lymphocytes were determined by staining the cells with FITC-CD4 or PE-cy5-CD8a. Flow cytometric analysis was performed on a FACScan system (BD Biosciences).

### LPS stimulation

For the in vitro stimulation test, whole blood was collected by removal of the eyeball bleed into EDTA-K2 vacutainer tubes at the indicated times after sham or CLP surgery. The blood was diluted 3 times with RPMI 1640 medium (1% glutamine, 1% pyruvate, 1% garamycine). Added 500 μl of the diluted blood to a 48-well culture plate and stimulated with LPS (100 ng/ml) for 1 h at 37 ℃ and under 5% CO_2_. After incubation, blood was centrifuged at 3000 rpm for 15 min and the level of TNFα in the plasma was examined by ELISA.

### Statistical analysis

Data are expressed as the mean ± SD, and statistically significant differences between mean values were determined by Student’s t test. Survival was analyzed using the Kaplan–Meier method, and statistical analyses were performed using the log-rank test. *p < 0.05, **p < 0.01, ***p < 0.001, and not significant, NS > 0.05.

## Supplementary information

Supplementary Information.

## Data Availability

The datasets generated during and analysed during the current study are available from the corresponding author on reasonable request.
