# Supplementary material for: Oroxylin A alleviates immunoparalysis of CLP mice by degrading CHOP through interacting with FBXO15
Source: Sci Rep. 2020 Nov 6;10:19272. doi: 10.1038/s41598-020-76285-x (PMC7648083; doi:10.1038/s41598-020-76285-x)
Supplement: Supplementary file 1 — Supplementary Information. [file 41598_2020_76285_MOESM1_ESM.doc]

**Oroxylin A alleviates immunoparalysis of CLP mice by degrading CHOP through interacting with FBXO15**

Zhaoxin Zhang^1^, Yun Wang^1^, Yating Shan^1^, Ri Zhou^1^ and Wu Yin^1*^

^1^The State Key Lab of Pharmaceutical Biotechnology, College of life Sciences, Nanjing, 210023, China

*Correspondence: [wyin@nju.edu.cn](mailto:wyin@nju.edu.cn)


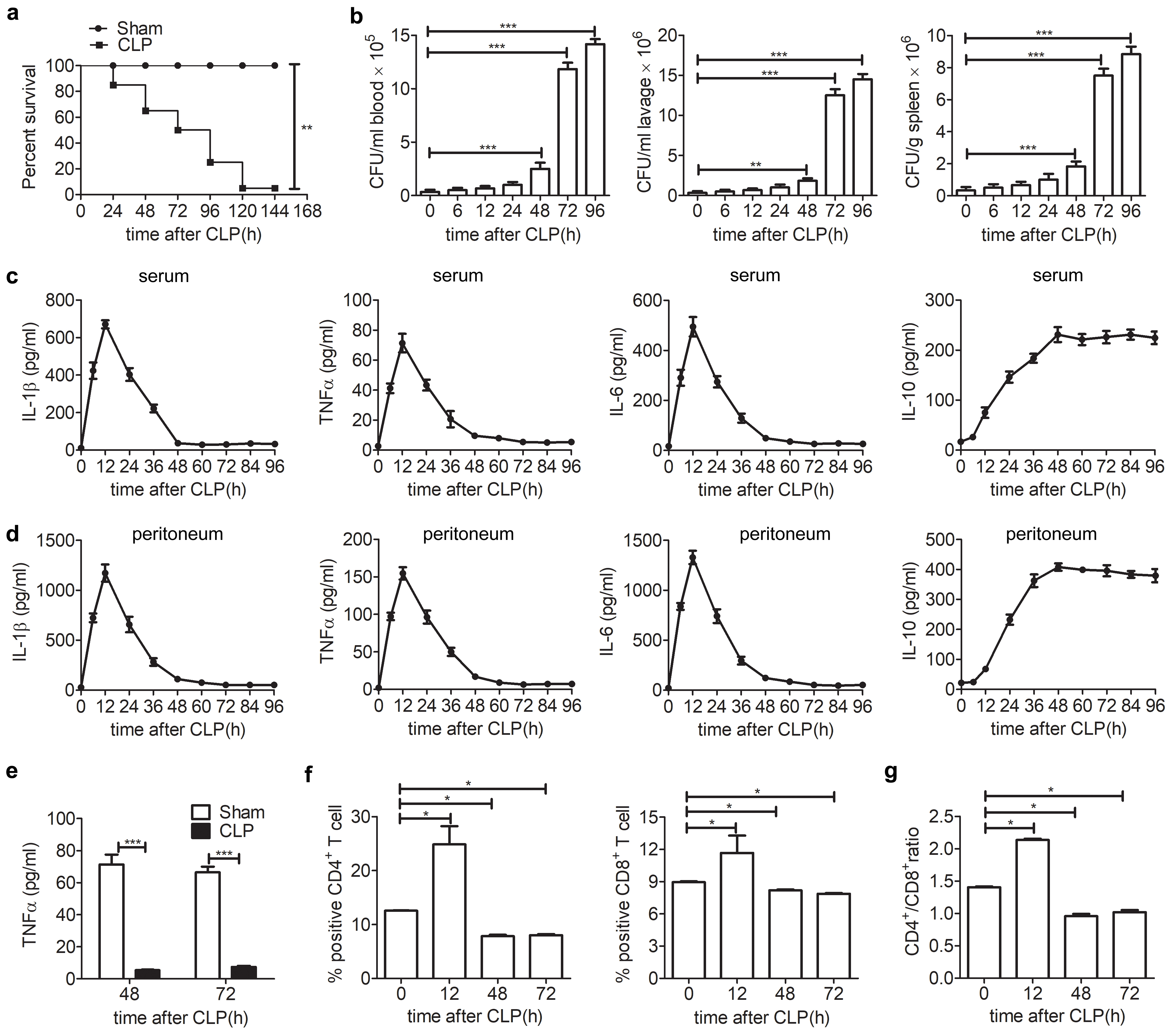


**Figure S1:** **Characteristics of the CLP model.**

(a) The mortality of CLP mice. Kaplan-Meier curves and the log-rank test were used to compare mortality rates (n = 20), **p < 0.01.

(b) The bacterial clearance of blood, peritoneum lavage and the spleen were examined at the indicated times after CLP (n = 6).

(c) The serum levels of IL-1β, TNFα, IL-6 and IL-10 were examined at the indicated times after CLP by ELISA (n = 6).

(d) The peritoneum lavage levels of IL-1β, TNFα, IL-6 and IL-10 were examined at the indicated times after CLP by ELISA (n = 6).

(e) Blood was collected at the indicated times after CLP and stimulated with LPS (100 ng/ml) for 1 h. The level of TNFα was examined by ELISA (n = 3).

(f) The percentages of CD4^+^ and CD8^+^ T cells in the spleen were examined at the indicated times after CLP by flow cytometry (n = 3). The flow histograms are presented in Supplementary Figs. S14 and S15.

(g) The ratio of CD4^+^/CD8^+^ T cells in the spleen was calculated according to the corresponding percentages of CD4^+^ and CD8^+^ T cells, respectively (n = 3).

The data are represented as the mean ± SD (b, e, f, g), *p < 0.05, **p < 0.01 and ***p < 0.001, student’s t-test.


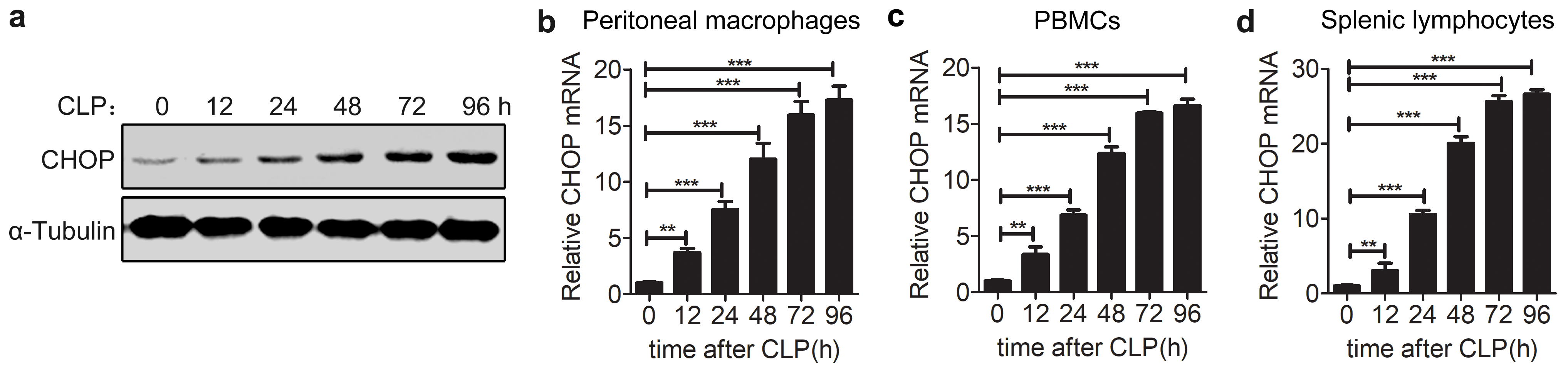


**Figure S2: CHOP is significantly upregulated and remains at high levels during immunoparalysis.**

(a, b) The protein and transcription levels of CHOP in peritoneal macrophages were examined at the indicated times after CLP by western blotting (a) and qPCR (b) (n = 6). The full-length blots are presented in Supplementary Fig. S14.

(c) The transcription levels of *chop* in PBMCs were examined at the indicated times after CLP by qPCR (n = 6).

(d) The transcription levels of *chop* in splenic lymphocytes were examined at the indicated times after CLP by qPCR (n = 6).

The data are represented as the mean ± SD, **p < 0.01 and ***p < 0.001, student’s t-test.


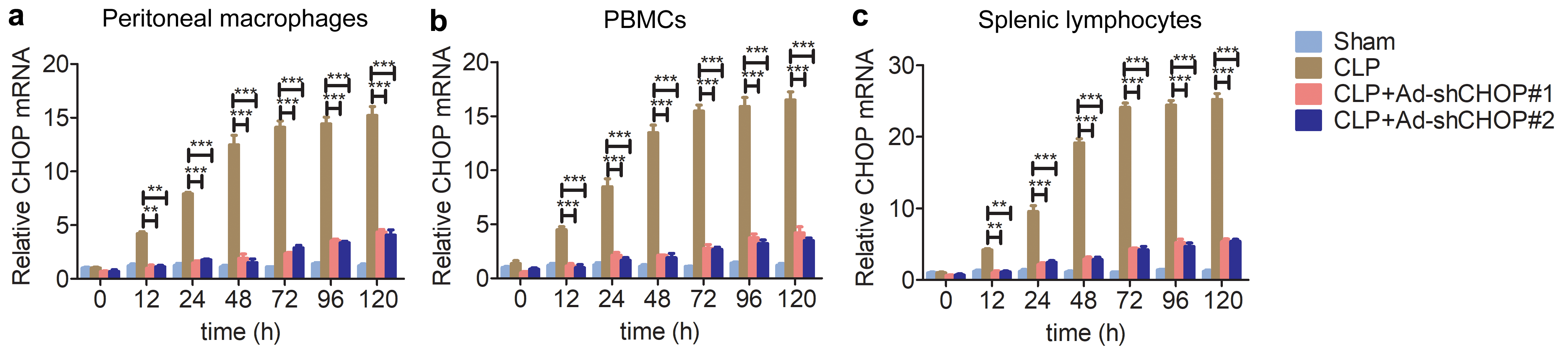


**Figure S3:** **The knockdown efficiency of shCHOP in CLP mice.**

Adenoviruses expressing shRNAs specifically targeting *chop* were intravenously injected into C57BL6 mice through the tail vein. After 7 days, CLP was performed. The mRNA levels of *chop* in peritoneal macrophages (a), PBMCs (b) and splenic lymphocytes (c) were examined at the indicated times after CLP by qPCR (n = 6).

The data are represented as the mean ± SD, **p < 0.01 and ***p < 0.001, student’s t-test.


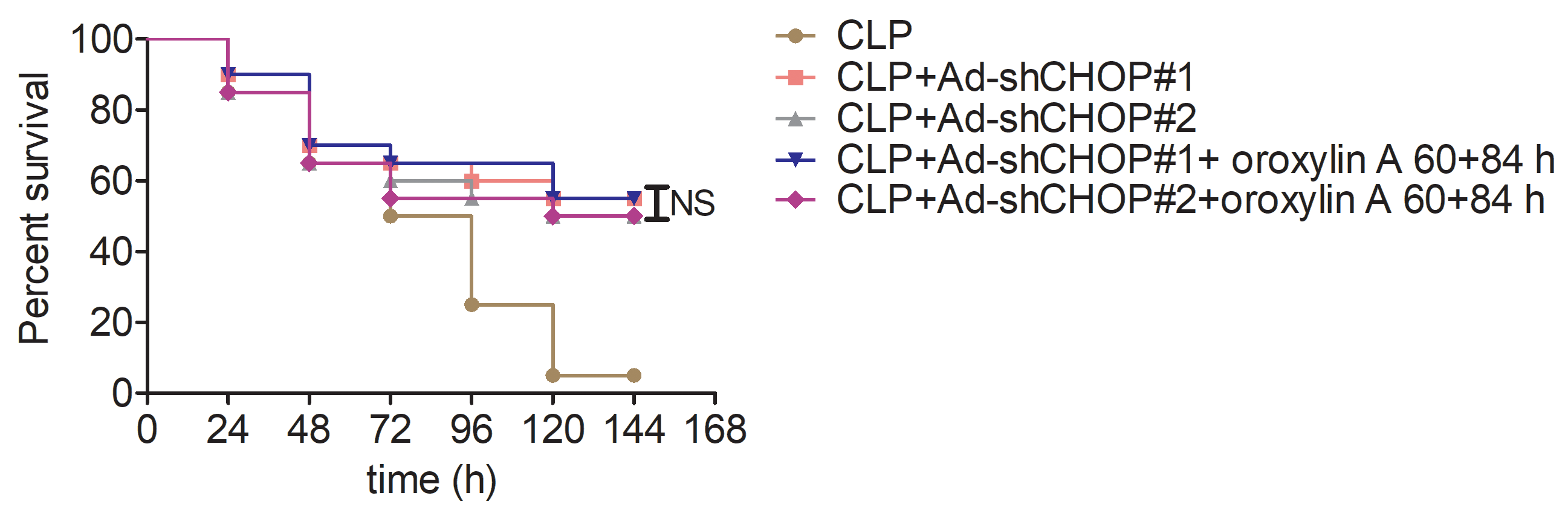


**Figure S4:** **The** **survival of CLP mice that is improved by knocking down *chop* is not further promoted by oroxylin A.**

Adenoviruses expressing shRNAs specifically targeting *chop* were intravenously injected into C57BL6 mice through the tail vein. After 7 days, CLP was performed and oroxylin A (30 mg/kg) was injected at 60 h and 84 h after the surgery. Kaplan-Meier curves and the log-rank test were used to compare survival rates (n = 20), NS > 0.05.


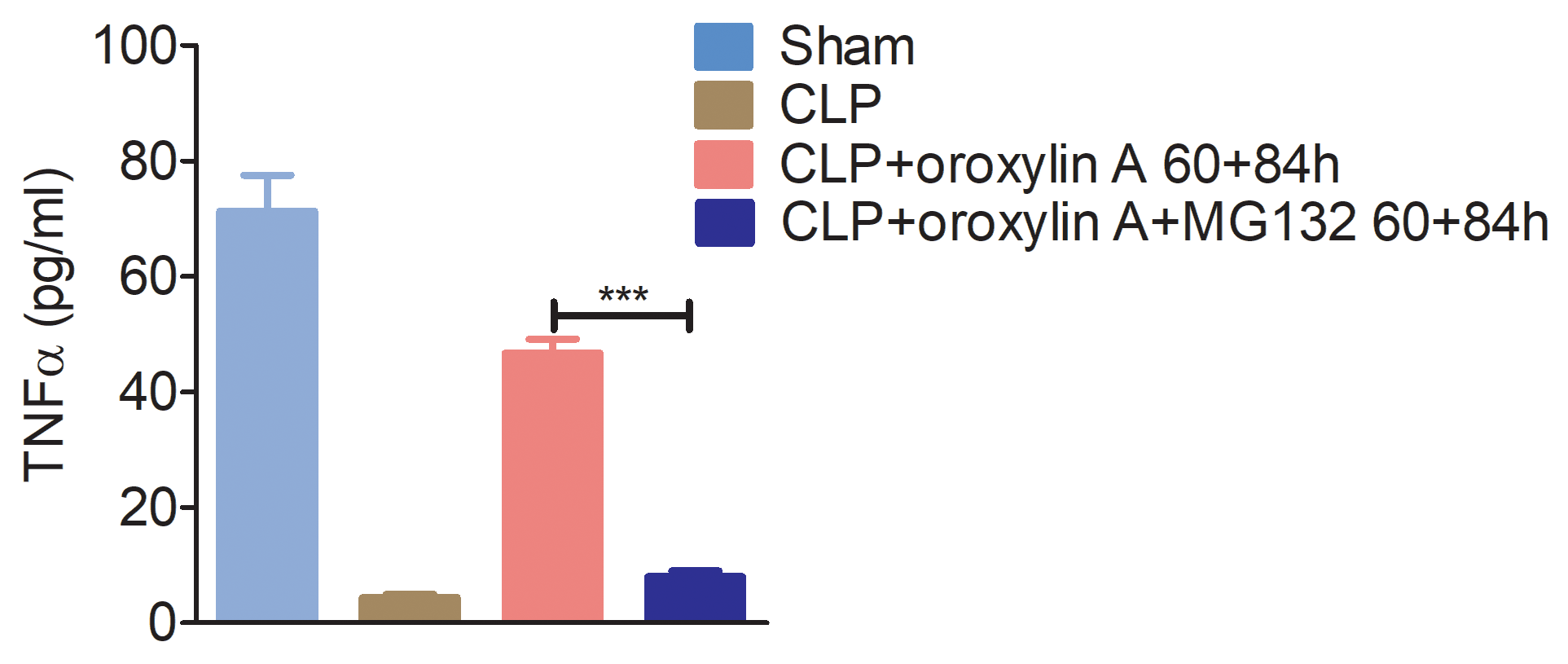


**Figure S5:** **The improvement effect of oroxylin A on TNFα production in the blood of CLP mice was inhibited by MG132.**

CLP mice were intraperitoneally injected with MG132 combined with oroxylin A at 60 h and 84 h after CLP. Blood was collected at 96 h after CLP and stimulated with LPS (100 ng/ml) for 1 h. The level of TNFα was examined by ELISA (n = 3).

The data are represented as the mean ± SD, ***p < 0.001, student’s t-test.


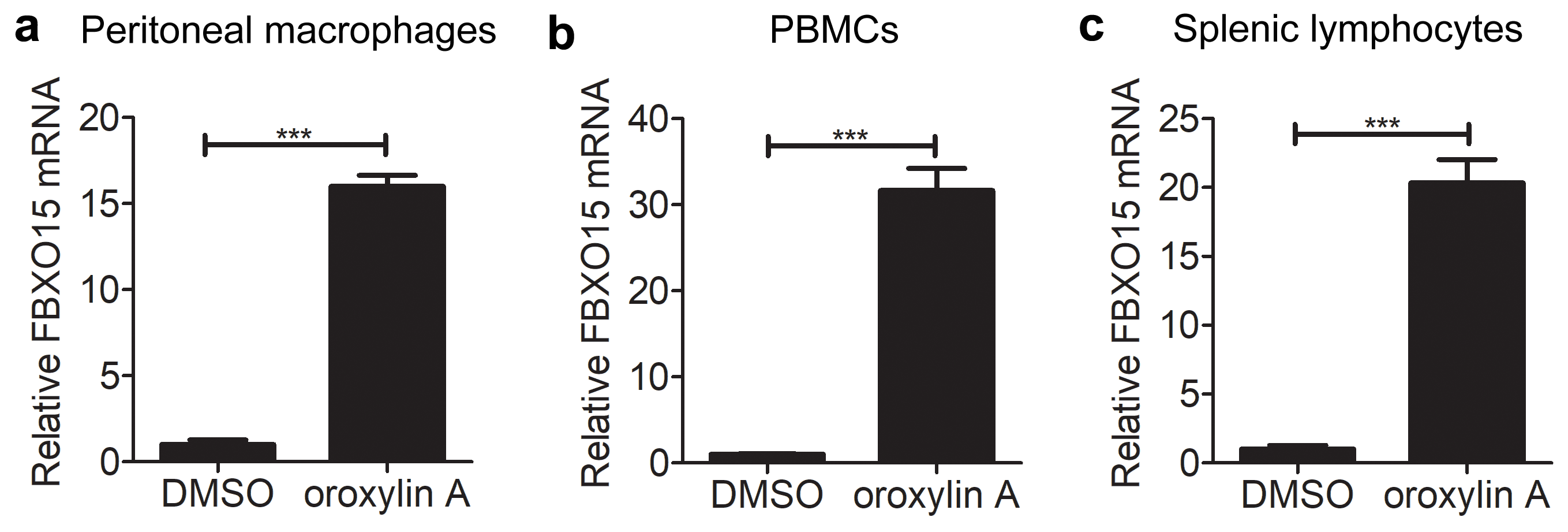


**Figure S6: Oroxylin A upregulates *fbx*o15 *in vitro.***

(a) Peritoneal macrophages were isolated from C57BL6 mice and treated with oroxylin A (40 μM) for 3 h. The mRNA expression of *fbx*o15 was examined by qPCR.

(b) PBMCs were isolated from C57BL6 mice and treated with oroxylin A (40 μM) for 3 h. The mRNA expression of *fbx*o15 was examined by qPCR.

(c) Splenic lymphocytes were isolated from C57BL6 mice and treated with oroxylin A (40 μM) for 3 h. The mRNA expression of *fbx*o15 was examined by qPCR.

The data are represented as the mean ± SD, ***p < 0.001, student’s t-test.


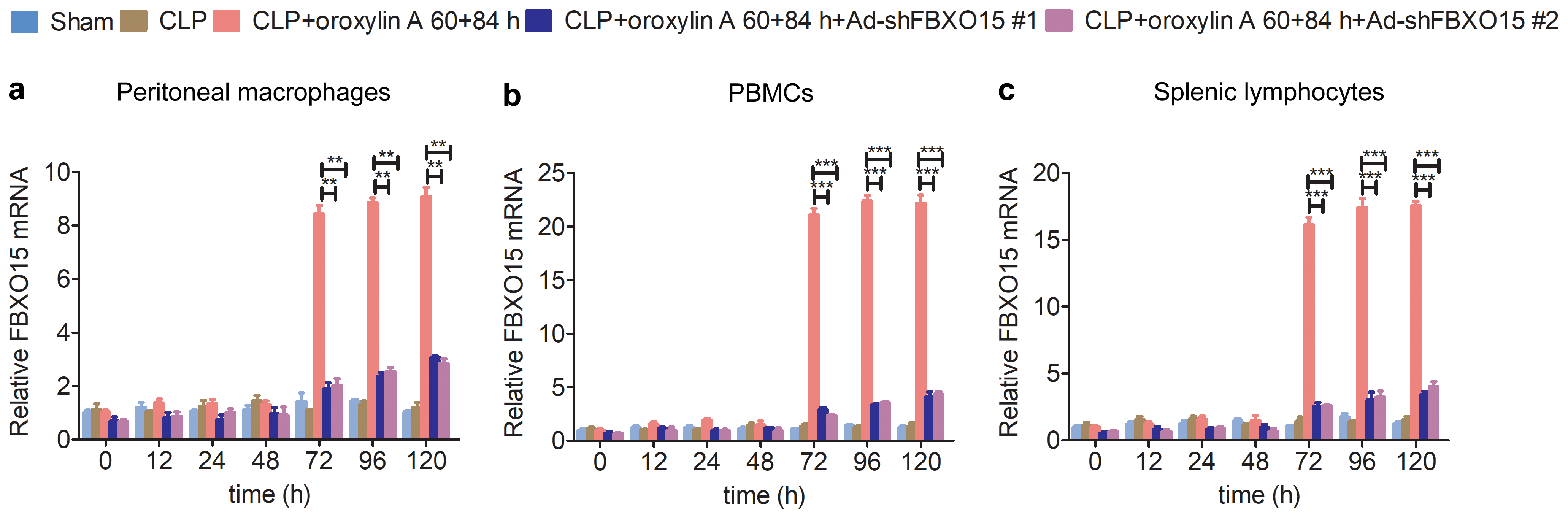


**Figure S7: The knockdown efficiency of shFBXO15 in CLP mice.**

Adenoviruses expressing shRNAs specifically targeting *fbx*o15 were intravenously injected into C57BL6 mice through the tail vein. After 7 days, mice underwent CLP surgery and were injected with oroxylin A (30 mg/kg) at the indicated times. The mRNA levels of *fbx*o15 in peritoneal macrophages (a), PBMCs (b) and splenic lymphocytes (c) were examined at 96 h after CLP by qPCR (n = 6).

The data are represented as the mean ± SD, **p < 0.01 and ***p < 0.001, student’s t-test.


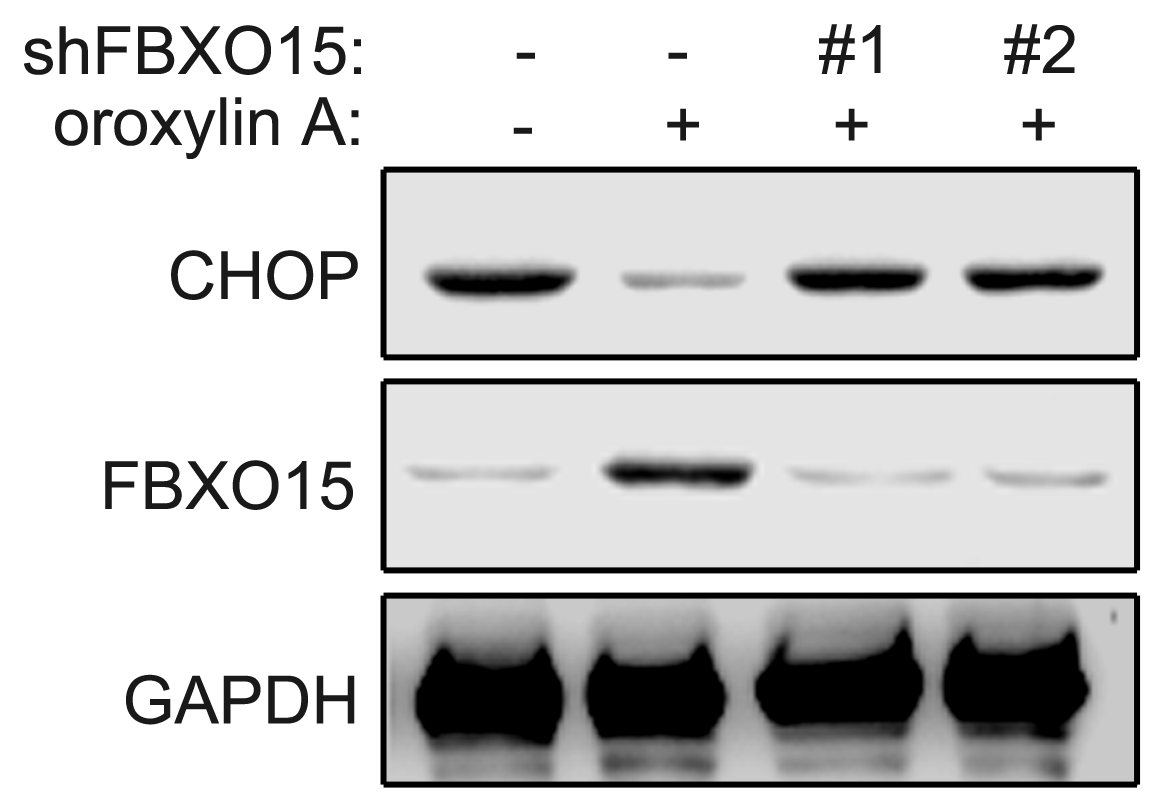


**Figure S8:** **Knockdown of *fbxo15* inhibits oroxylin A's downregulation of CHOP protein during immunoparalysis.**

Adenoviruses expressing shRNAs specifically targeting *fbxo15* were intravenously injected into C57BL6 mice through the tail vein. After 7 days, CLP was performed and oroxylin A (30 mg/kg) was injected at 60 h and 84 h after CLP. The protein level of CHOP in peritoneal macrophages was examined at 96 h after CLP by western blotting. The full-length blots are presented in Supplementary Fig. S14.


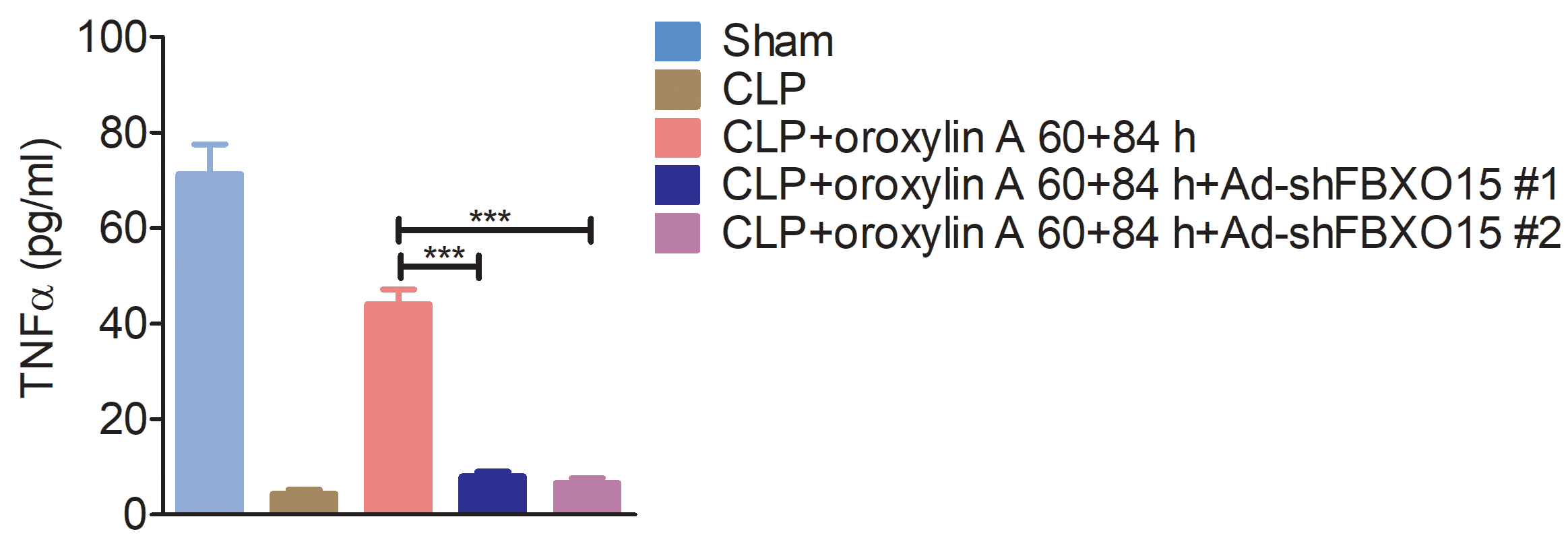


**Figure S9: The improvement effect of oroxylin A on TNFα production in the blood of CLP mice was inhibited by knockdown of *fbxo15*.**

Adenoviruses expressing shRNAs specifically targeting *fbxo15* were intravenously injected into C57BL6 mice through the tail vein. After 7 days, CLP was performed and oroxylin A (30 mg/kg) was injected at 60 h and 84 h after CLP. Blood was collected at 96 h after CLP and stimulated with LPS (100 ng/ml) for 1 h. The level of TNFα was examined by ELISA (n = 3).

The data are represented as the mean ± SD, ***p < 0.001, student’s t-test.


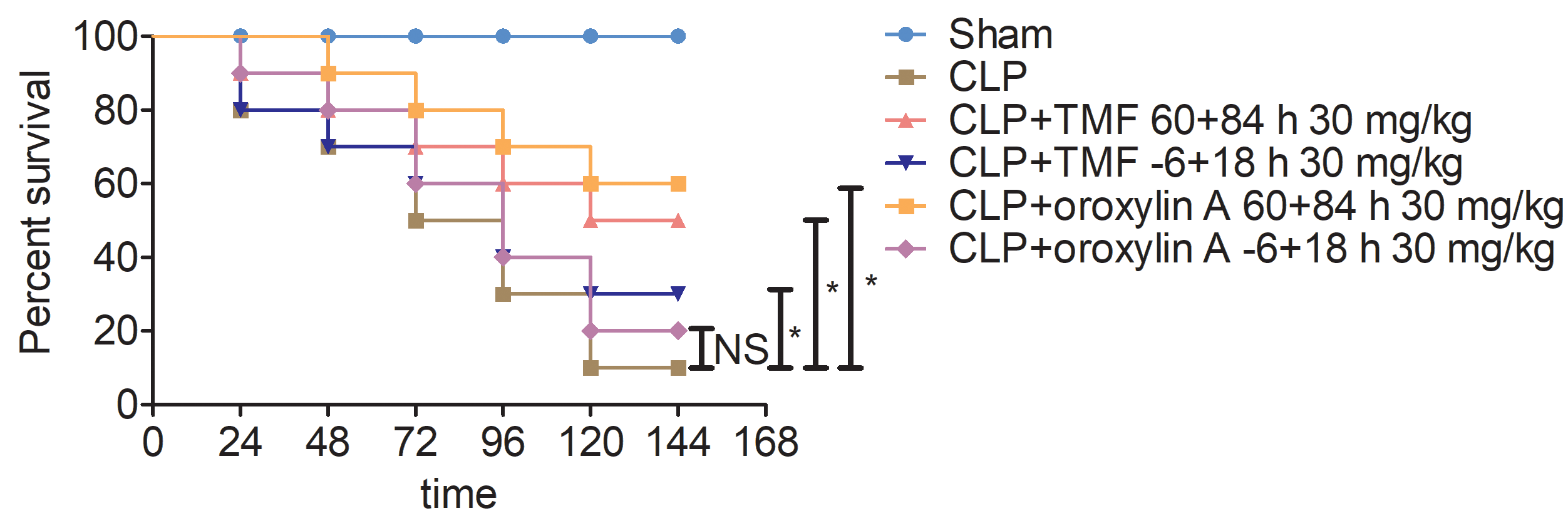


**Figure S10: The effect of TMF on the survival of CLP mice.**

Mice were injected with TMF or oroxylin A at the indicated times and the effect of them on the mortality of CLP mice was detected (n = 10). Kaplan-Meier curves and the log-rank test were used to compare mortality rates, *p < 0.05.


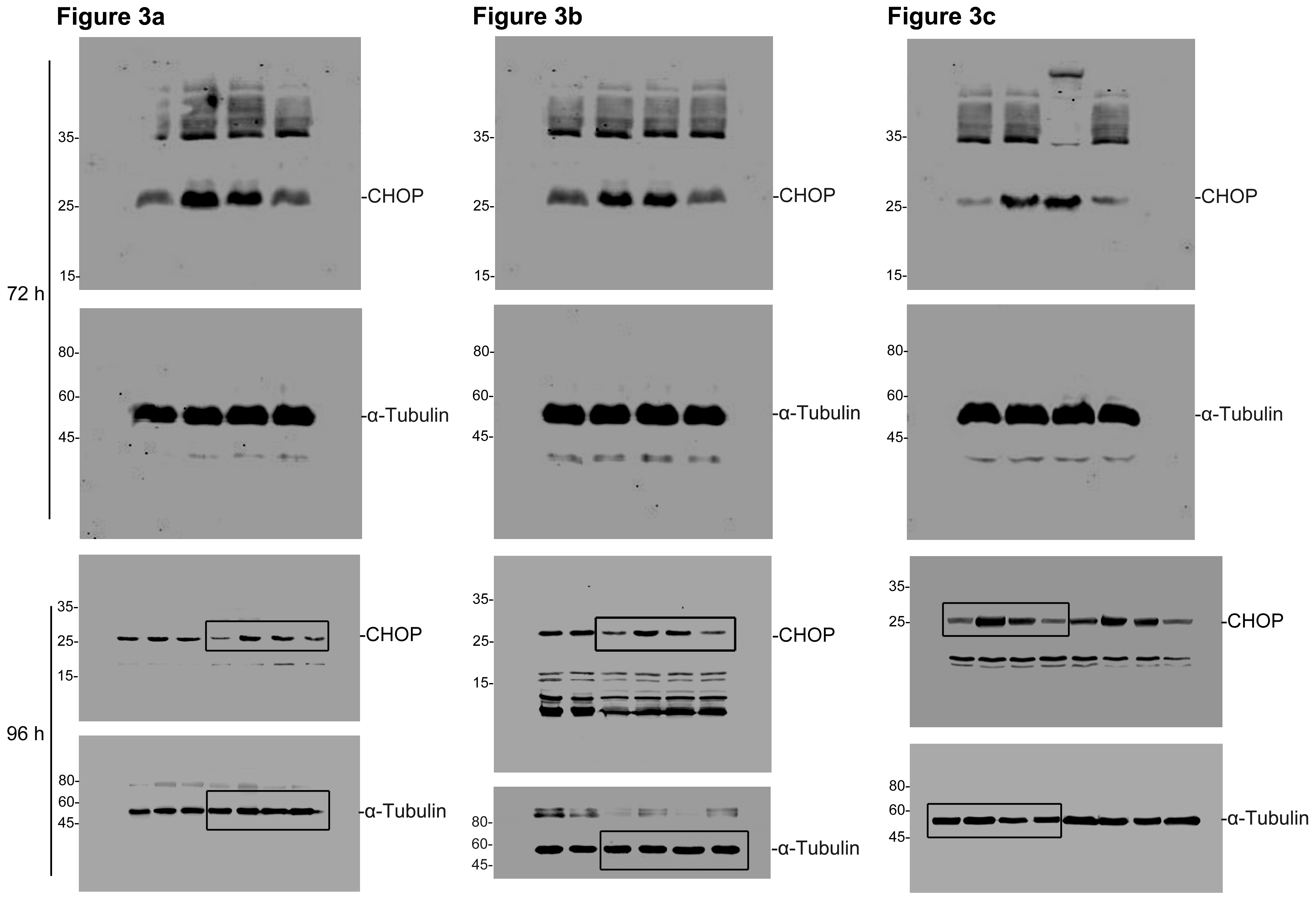


**Figure S11:** **The full-length blots of Fig. 3.**


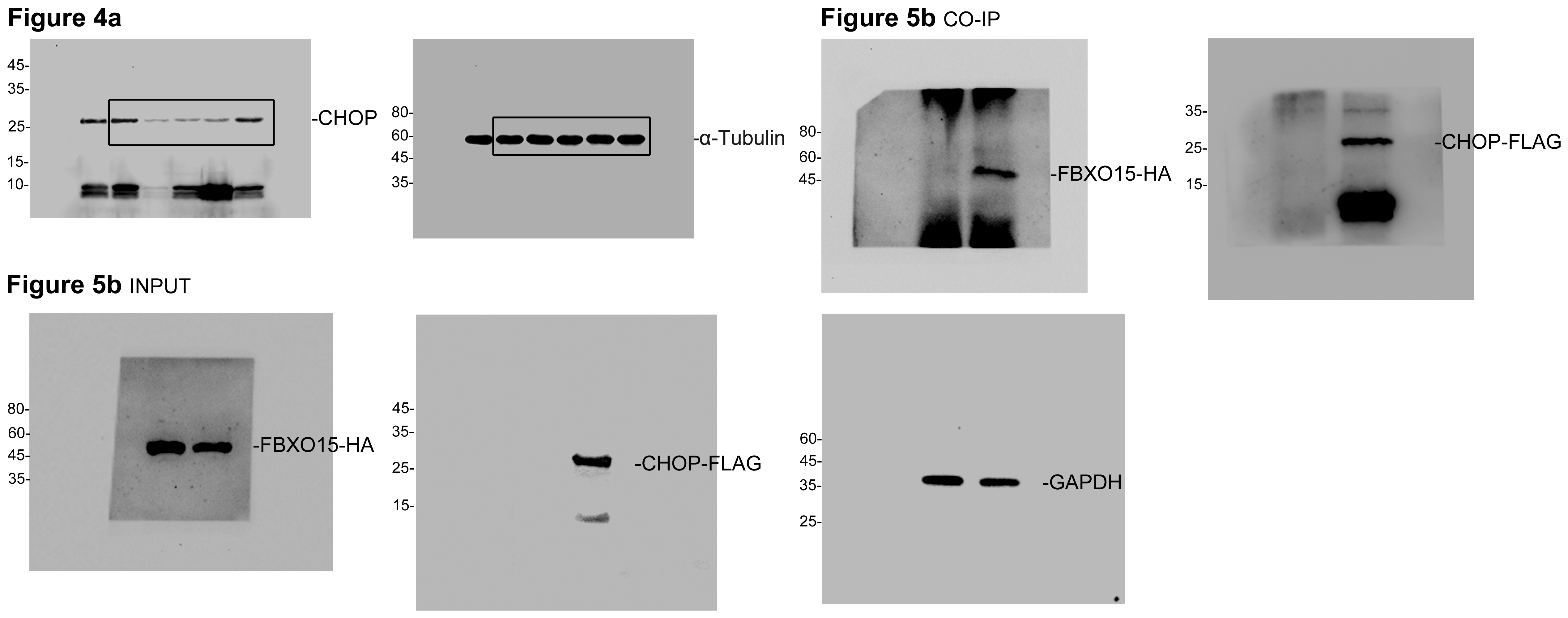


**Figure S12:** **The full-length blots of Figs. 4-5.**


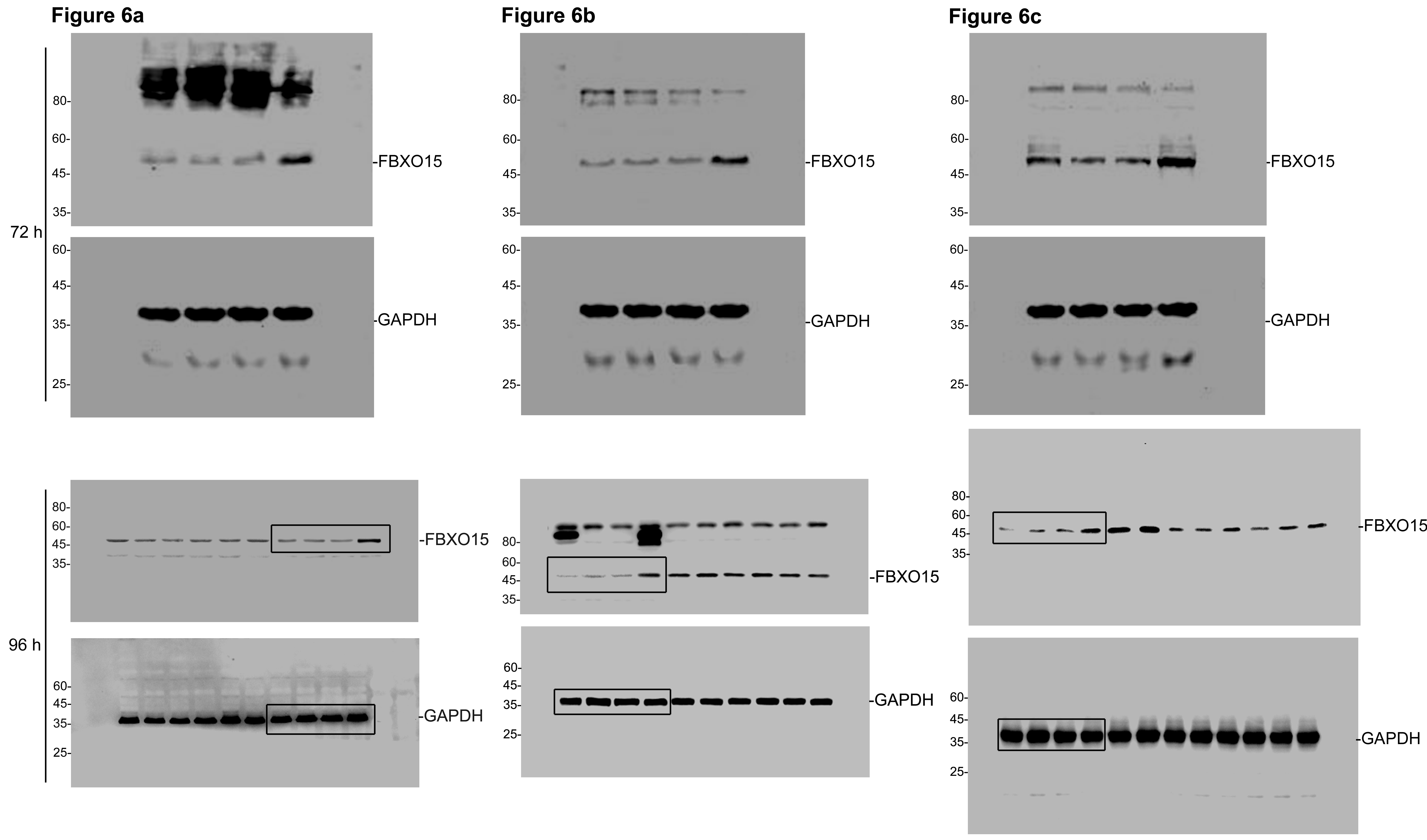


**Figure S13:** **The full-length blots of Fig. 6.**


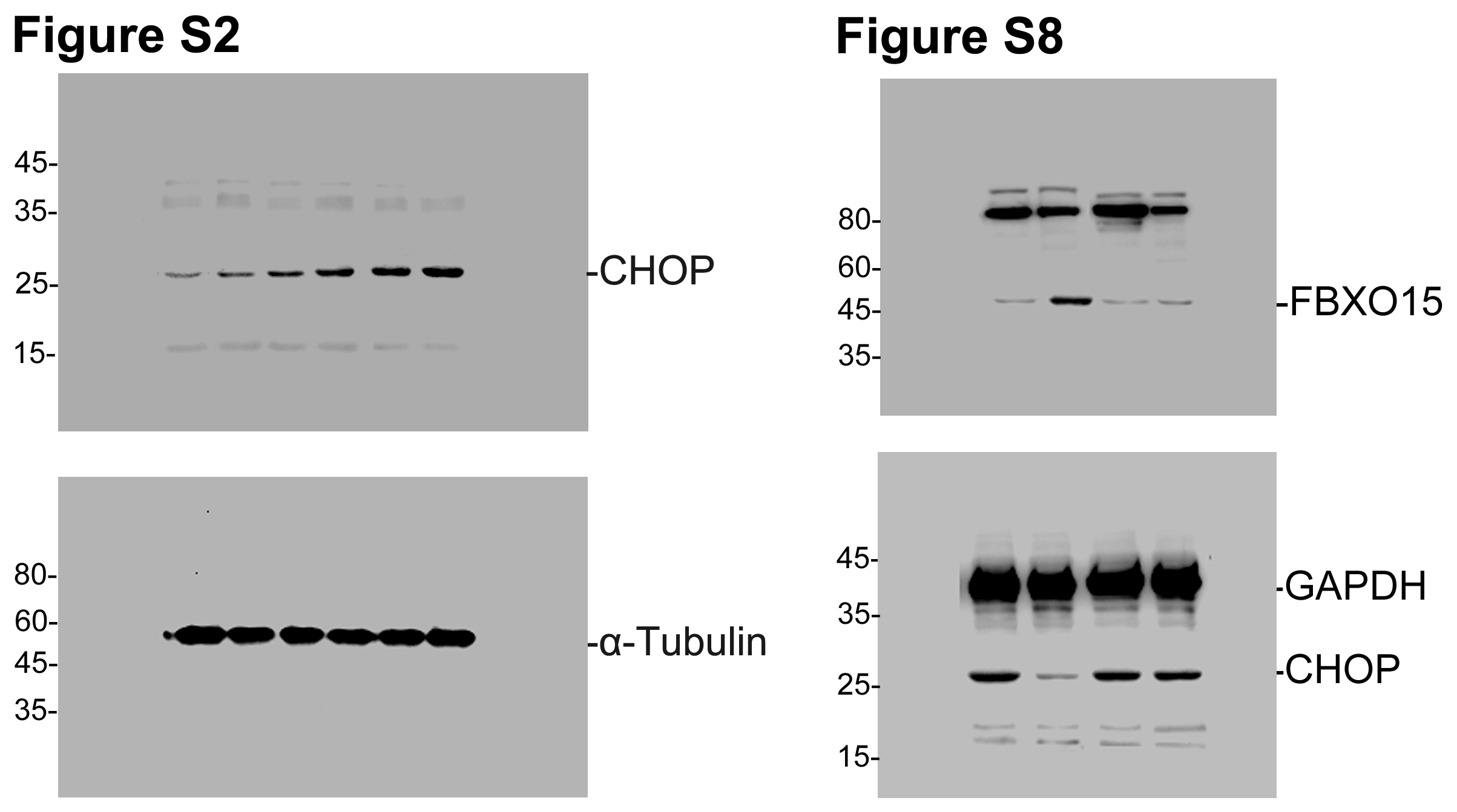


**Figure S14:** **The full-length blots of Supplementary Figs. S2 and S8.**


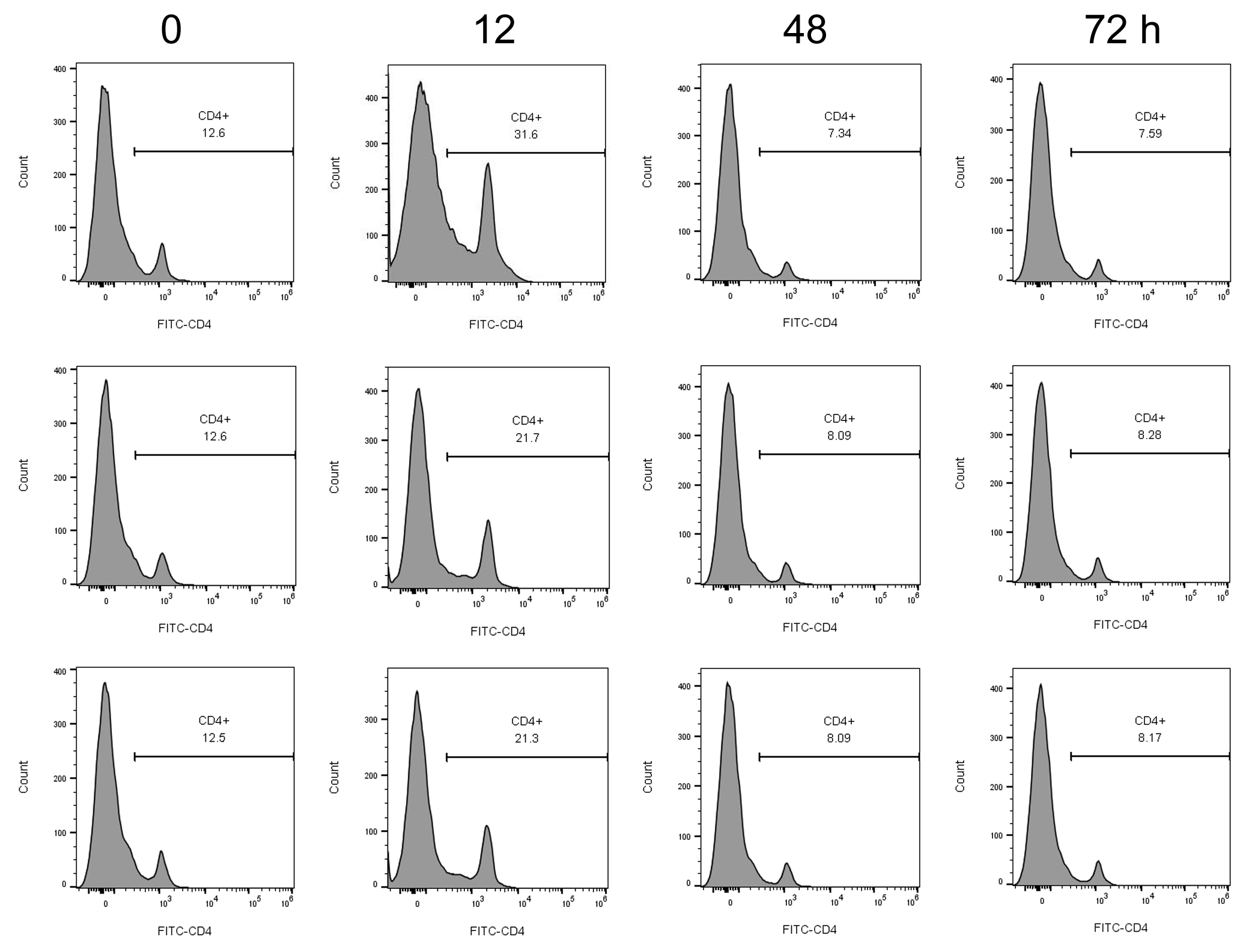


**Figure S15: The flow histograms of Supplementary Fig. 1 (CD4^+^).**


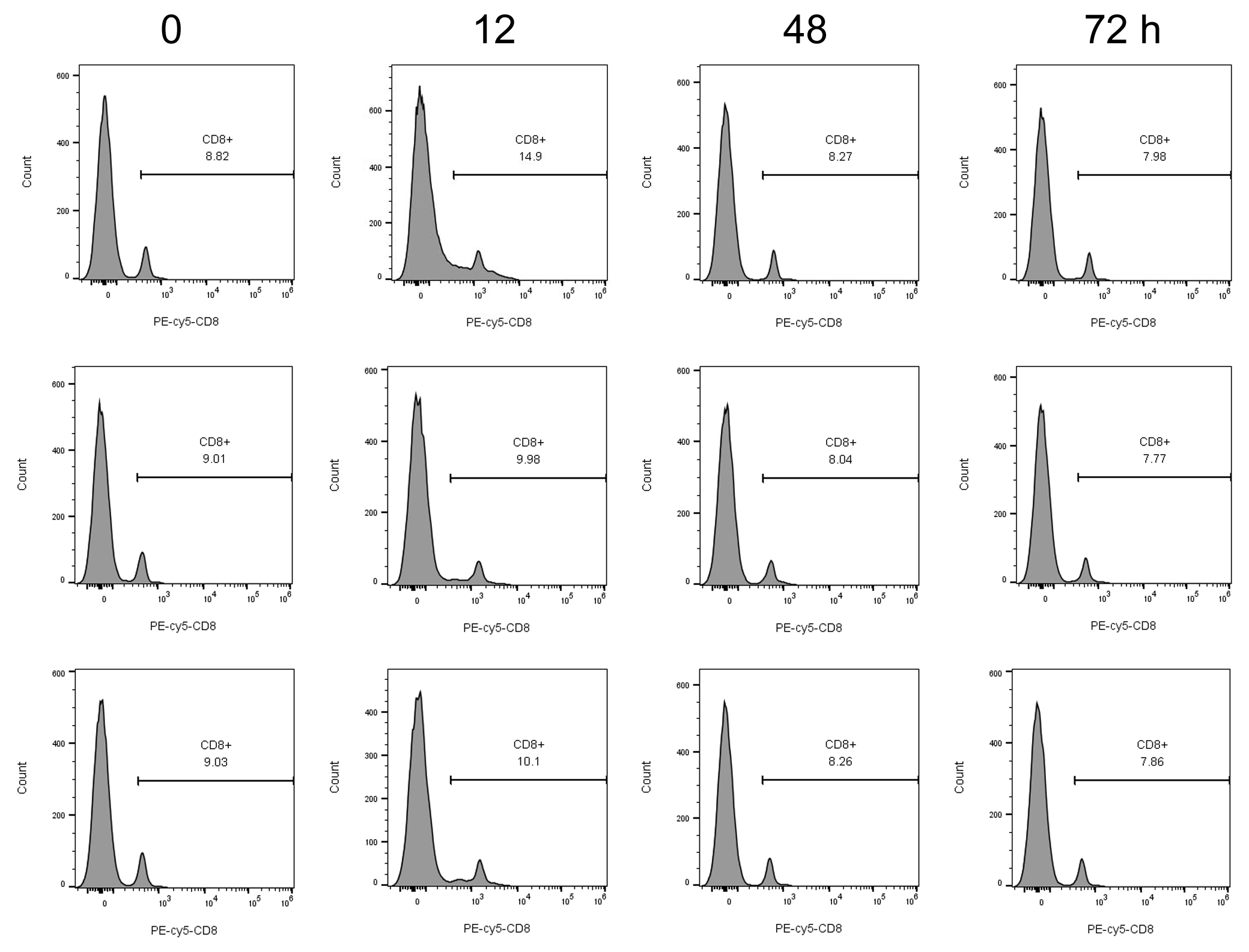


**Figure S16: The flow histograms of Supplementary Fig. 1 (CD8^+^).**


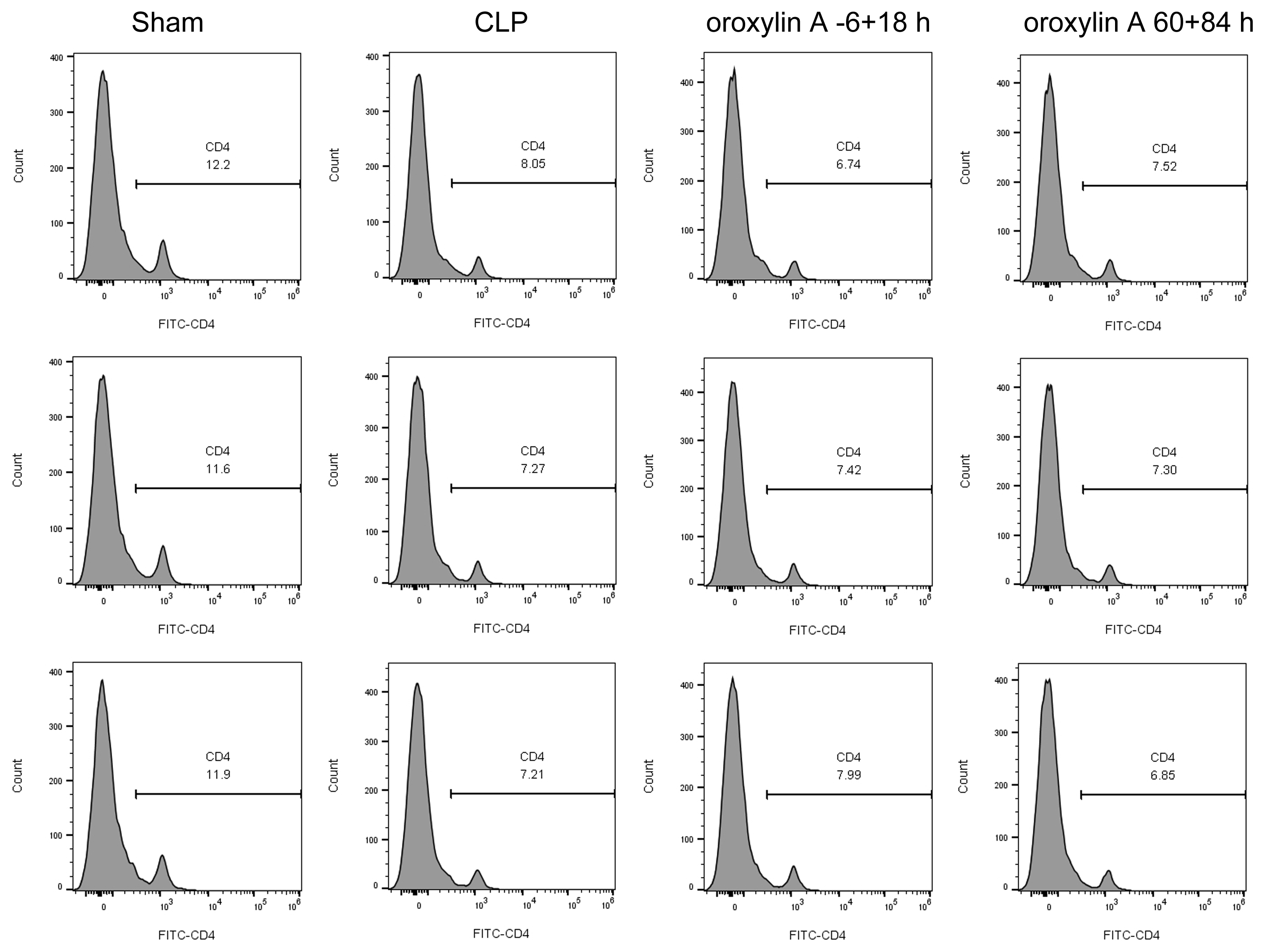


**Figure S17: The flow histograms of Fig. 1 (CD4^+^).**


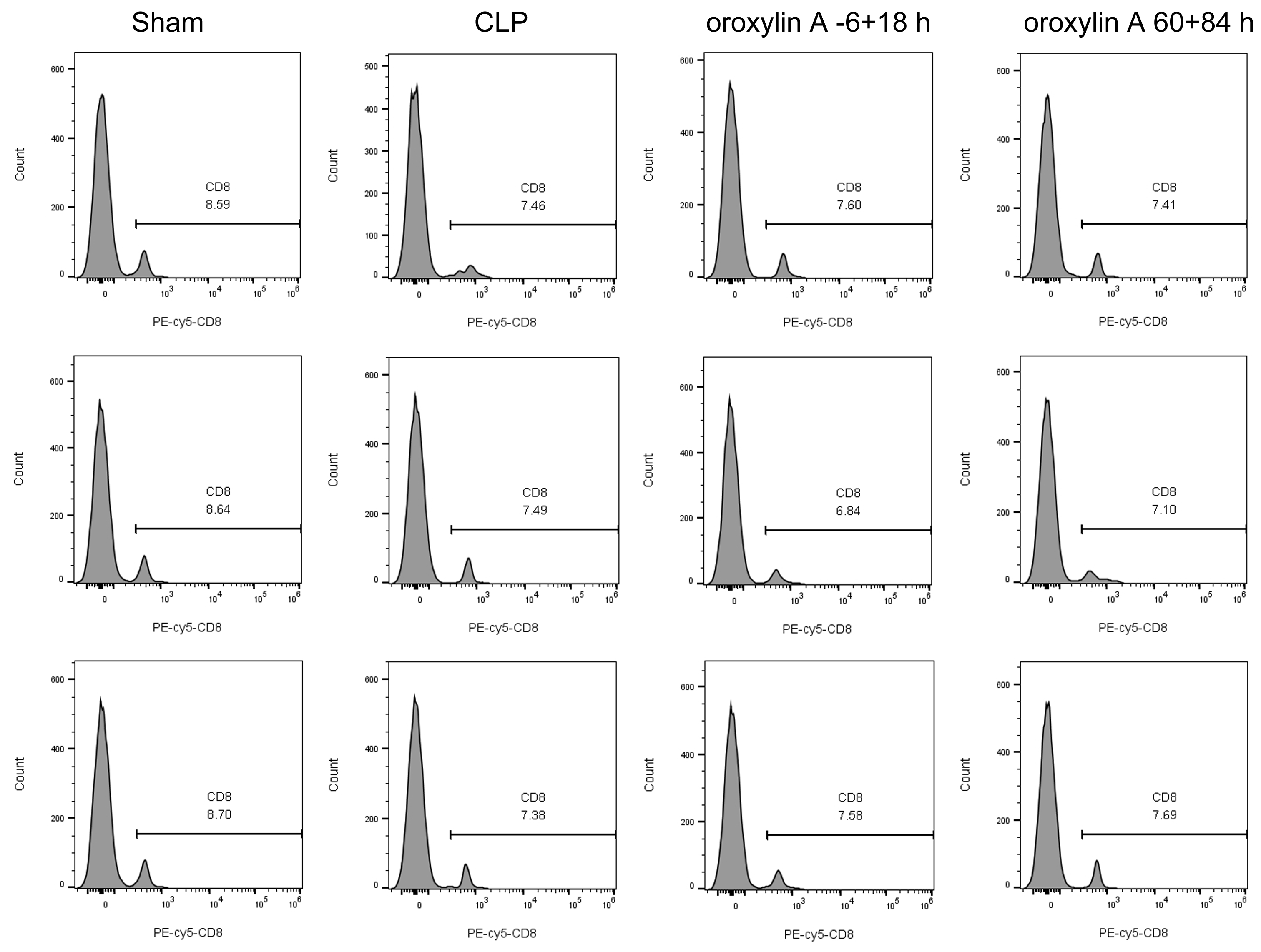


**Figure S18: The flow histograms of Fig. 1 (CD8^+^).**


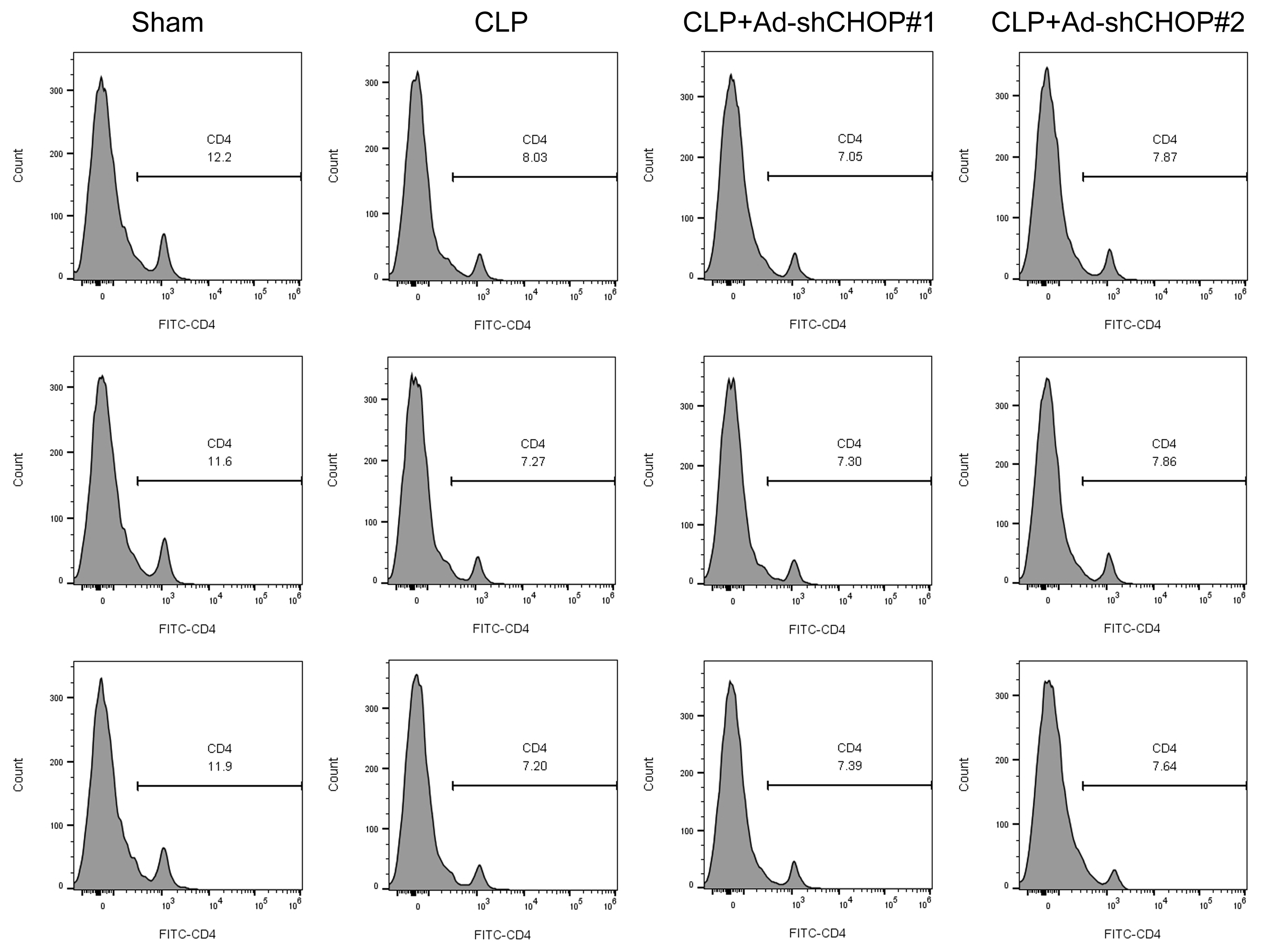


**Figure S19: The flow histograms of Fig. 2 (CD4^+^).**


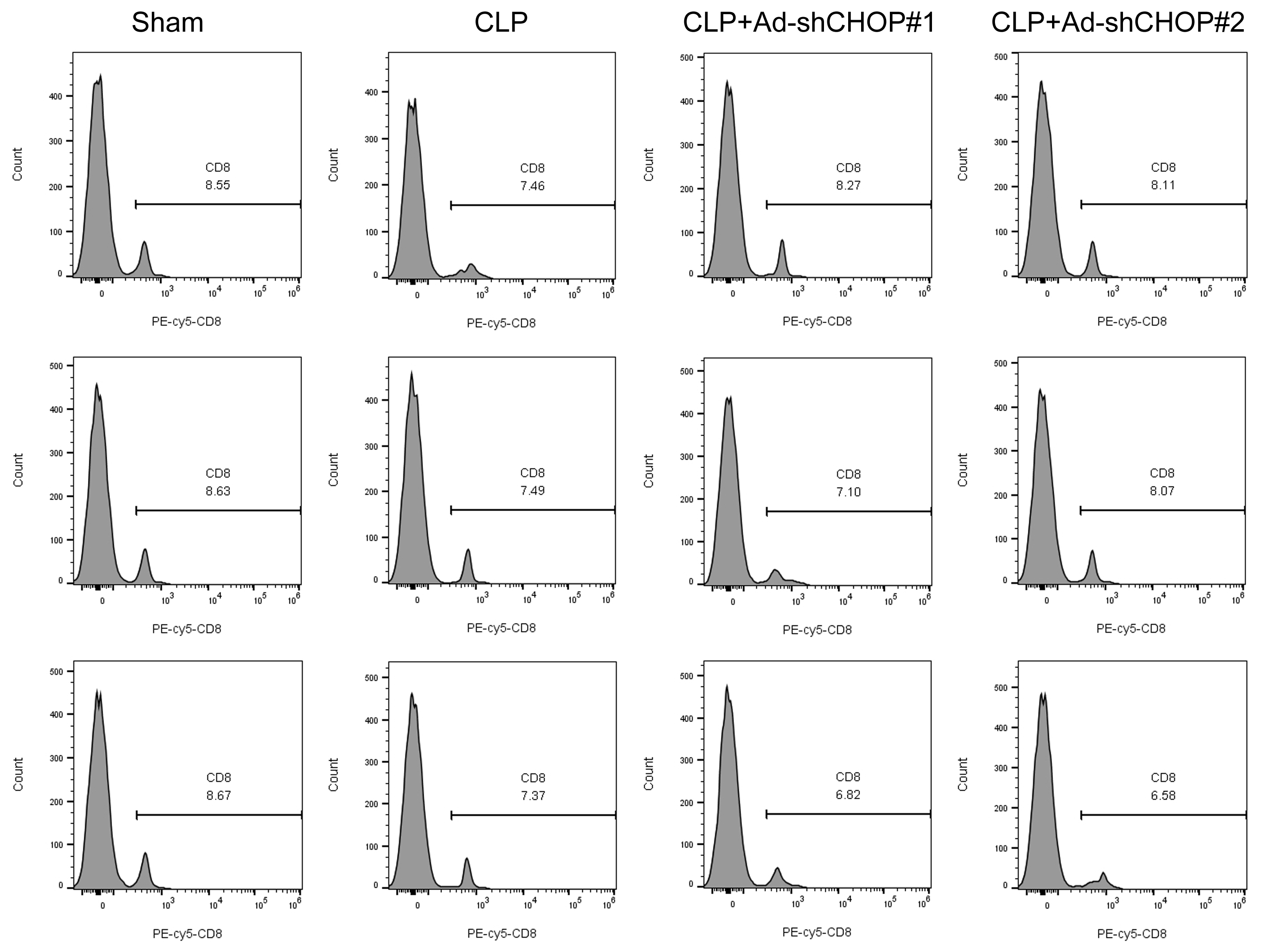


**Figure S20: The flow histograms of Fig. 2 (CD8^+^).**

**Table S1 Primer sequences**

| shCHOP #1 | CCGGGGAAGAACTAGGAAACGGACTCGAGTCCGTTTCCTAGTTCTTCCTTTTT |
| --- | --- |
| shCHOP #2 | CCGGGGGCTCTGATCGACCGCATCTCGAGATGCGGTCGATCTGAGCCCTTTTT |
| shFBXO15 #1 | CCGGCAGTTTTAGAAGTAAGTTTGTTACTCGAGTAACAAACTTACTTCTAAAACTGTTTTT |
| shFBXO15 #2 | CCGGTCGGAAATCTTGGTGAAGATACTCTCGAGAGTATCTTCACCAAGATTTCCGATTTTT |
| CHOP-F | GCTGAGTCCCTGCCTTTC |
| CHOP-R | GCTTTGGGATGTGCGTGT. |
| FBXO15-F | ATCGGAAATCTTGGTGAA |
| FBXO15-R | AGACTTGTGGCTGTTTCC |
| pGBKT7-CHOP-F | CCGGAATTCATGGCAGCTGAGTCCCTGCC |
| pGBKT7-CHOP-R | CGCGGATCCTCATGCTTGGTGCAGGCTGA |
| pGADT7-FBXO15-F | CCGGAATTCATGGCGAGCGGACGCGGTCG |
| pGADT7-FBXO15-R | CGCGGATCCTTATTGCCTCCCAAACCATT |
| pCMV-FLAG-C-CHOP-F | CCGGAATTCTGGCAGCTGAGTCCCTGCC |
| pCMV-FLAG-C-CHOP-R | CGGGGTACCTCATGCTTGGTGCAGGCTGA |
| pCMV-HA-C-FBXO15-F | CGGGGTACCATGGCGAGCGGACGCGGTCG |
| pCMV-HA-C-FBXO15-R | AAGGAAAAAAGCGGCCGCTTATTGCCTCCCAAACCATT |
